# Aging and trace elements in human coronal tooth dentine

**DOI:** 10.1038/s41598-020-66472-1

**Published:** 2020-06-19

**Authors:** Ana C. Fernández-Escudero, Isabel Legaz, Gemma Prieto-Bonete, Manuel López-Nicolás, Antonio Maurandi-López, María D. Pérez-Cárceles

**Affiliations:** 1 Department of Legal and Forensic Medicine, Biomedical Research Institute (IMIB), Regional Campus of International Excellence “Campus Mare Nostrum”, Faculty of Medicine, University of Murcia, Murcia, Spain; 2 Department of Dermatology, Stomatology, Radiology and Physical Medicine, University of Murcia, Murcia, Spain; 3 Department of Didactics of Mathematical and Social Sciences, University of Murcia, Murcia, Spain

**Keywords:** Biomarkers, Medical research, Translational research

## Abstract

Teeth are a fundamental tool in forensic odontology for identification in a legal context of those individuals who cannot be identified visually or by other means. Dentine presents physiological exchanges of in trace elements after a period of mineralization and several factors can affect its concentration. The aim of this study was to investigate the concentration of 25 trace elements in the coronal dentine according to sex and type of tooth to determine their relationship with age. A total of 25 trace elements were analyzed in 150 human coronal dentine. Teeth were classified into three age groups, sex and tooth type. The trace elements were grouped as potentially toxic or essential. Inductively Coupled Plasma-Mass Spectrometry and Atomic Emission Spectroscopy were used. The toxic and essential elements were detected in the following order of concentration: Al > Pb > Sn > Li > As > Cd and Ca > P > Mg > Na > S > K > Sr > Zn > Ba > Fe > B > Ti > Mn > Cr > Ni > Cu > Co > Se > V. Our findings show an increase in the concentration of toxic (Pb, Li and Sn) and essential (B, Ba, K, Sr, S and Mg) elements in coronal dentin related to the age of the teeth, regardless of sex. The concentrations of Pb and K in dentin of molars and premolars are the elements that best relate their variations with age. In view of our results, the analysis of these trace elements in dentin in combination with other types of techniques could be established as an element to consider in age dating studies in different forensic situations.

## Introduction

Teeth and oral structures are a fundamental tool in forensic odontology for identification in a legal context of those individuals who cannot be identified visually or by other means^1^. The hardness of dental structures allows teeth to be preserved over time, being resistant to decomposition and high temperatures, making them very useful in postmortem analysis and essential for the identification of human remains^2–8^. Recent studies have investigated the relationship between the trace elements in human teeth and pollution^9–11^ and as a bio-indicator of the nutritional status or disease^12^. Different methods are continuously being developed to estimate dental age using dental tissues, and histological, radiographic and biochemical procedures, but none is considered very accurate^13–19^. Dental age determination is becoming important in cases of people without any identity or bone remains^20,21^, competitive sports with age limits^22^, and also to estimate dental age among under aged commercial sex workers with the intention of rehabilitation, and in paleodontological studies^20,23^. Teeth, then, are an important adjunct in forensics.

The biomineralization of teeth is a dynamic, complex and lifelong process through which inorganic nanocrystal precipitations occur within the organic matrices to form hybrid biological tissues^24^. Teeth have the ability to incorporate many chemical elements into their structure, the most abundant being calcium, phosphorus, magnesium and sodium^24–27^. Dental structures contain a wide variety of trace elements but most studies analyze the concentration of trace elements in dental enamel^28^.

Dentine is surrounded by enamel and cementum, and is not affected by the oral environment^29^. It also presents a physiological exchange of elements after a period of mineralization, contrary to that which occurs in enamel^30^. Moreover, dentine is characterized by having a non-active metabolism of elements after dentine formation is complete and the mobility of the substances it contains is low compared with other hard tissues, providing a permanent, cumulative and solid record of past and/or recent heavy metal environmental exposure^10,31–34^.

Many studies show that there are several factors that can affect the concentration of trace elements in whole-teeth, when making correlations between samples and environmental conditions, life habits (diet and tobacco), region of origin, caries, diseases, such as diabetes or high blood pressure^35–42^. However, the concentration of trace elements in dental dentine may be affected by sex and show different degrees of sensitivity according to age, but dates on the trace elements found in tooth dentine is scarce^33,43^.

A study found that many dentin elements increase with age until the age of fifty^33^. It is believed to be attributable to the fact that dentin contains a significant quantity of collagen fibers and that, after the elements that have affinity for collagen fiber are processed in the body and the accumulated dose increased, concentrations increase in relation to age.

The aim of this study was to investigate the concentration of twenty-five trace elements (toxic and essential) in the coronal dentine of healthy teeth according to sex and type of tooth to determine any relationship between the accumulation of trace elements and age and, consequently, to establish the usefulness of dental dentine as a long-term dental element suitable for age studies in forensic situations.

## Patients and methods

### Studied populations

A total of 150 healthy permanent teeth were extracted from different patients due to orthodontic treatment or therapeutic interventions between 2016 and 2018 in the University Dental Clinic, University of Murcia (Spain). All the teeth belonged to patients of Spanish nationality, residing in Murcia, a Mediterranean city in the southeast of Spain (Table 1) whose predominant diet was the Mediterranean diet. The following exclusion criteria were established: patients with occupational exposure to metals (heavy metal industry and gas-chemistry in particular); smoking in the past and present; pregnancy and lactation; acute infections; acute surgical diseases and trauma; chronic disease, metal implants; receive trace element preparations today; vegetarian diet. Lithologically, the area of Murcia is characterized by calcareous, dolostone, marl and metamorphic materials, primarily phyllites. The predominant soils in the area of Murcia have a carbonated composition, primarily xerosols, litosols, regosols, and fluvisols^44–46^.

**Table 1:** Description of the teeth samples used in this study.

	Patients		
	Total, N = 150	Men, N = 63, (42%) n (%)	Women, N = 87, (58%) n (%)
Age (years)*	40.37 ± 1.55	48.06 ± 18.98	34.23 ± 16.54
Age groups (years)			
Group I (<30)	56 (37.3)	15 (24.0)	41 (47.1)
Group II (30–50)	45 (30.0)	13 (20.0)	32 (36.8)
Group III (>50)	49 (32.7)	35 (56.0)	14 (16.1)
Tooth type			
Molar	109 (72.6)	45 (71.5)	64 (73.6)
Premolar	41 (27.4)	18 (28.5)	23 (26.4)

N, total number of each tooth; n (%), number and percentage of tooth in each group; SE, Standard error. International nomenclature of World Dental Federation (https://www.fdiworlddental.org/) was used to classification each dental piece. *Median age ± SE.

The different dental pieces analyzed in this study were classified following the nomenclature of the World Dental Federation^47^ and include a total of 109 molars and 41 premolars. The median age of the total cohort was 40.37 ± 1.55 (years ± SEM) with an age range between 18 to 88 years. Of the dental pieces analysed 42% corresponded to men (n = 63) and 58% to women (n = 87). Teeth in poor condition (caries, much erosion or abrasion, cracks, cavitations, restorations or defects) were excluded.

For the study, the teeth were divided into three age groups and receiver operation characteristic (ROC) curves were used to find an optimal cut-off value for each group: Group 1 (<30 years), n = 56; Group 2 (30–50 years), n = 45 and Group 3 (>50 years), n = 49. A range of optimal values was obtained between 29.8 and 49.7 years, with sensitivity of 0.718–0.498, and specificity of 0.687–0.795. The final cut-off values of 30 and 50 years was chosen.

Informed consent was obtained from all the participants included in the study. The study protocol was approved by the institutional ethics committee in accordance with the Helsinki Declaration of 2000 and the protocol was approved by the Ethical Research Committee of Murcia University (ID2035/2018).

### Sample preparation

All the teeth were classified and washed in distilled water, air-dried and frozen at −20 °C after extraction until use. All the instruments used during the teeth preparation phase were washed with 10% nitric acid and then rinsed with distilled water and dried in a laboratory incubator (70 °C). Prior to analysis, the teeth were sterilized in a steam autoclave at 134 °C and 30 psi for 3 min. To remove the soft tissues, gums and blood the teeth were soaked for 2 h in 30% hydrogen peroxide with occasional agitation and washed three times with Milli-Q quality water and decoronated^48,49^, since this allows the crown to be separated from the root with the minimum risk of contamination.

Enamel, cementum and the pulp were removed, obtaining the coronal dentine block, which was again cleaned in an ultrasonic bath of ultrapure water (18.2 MΩ^−cm^ resistivity) and dried in an oven at 60 °C for 2 h^50^. Once dry, each coronal dentine block was ground in an agate mortar, and 0.2 g was weighed on a precision balance. Digestion was performed using a digester (Microwave Milestone Model Ultrawave). The sample was placed in a Teflon tube with 4 ml of a high purity 65% nitric acid (Suprapur, Merck), 4 ml of Milli-Q water and 2 ml of 30% hydrogen peroxide. All the chemical reagents used during the analysis and preparation had been awarded quality certificates. After the digestion process, the resulting solution was transferred to a volumetric flask and was flushed with distilled water and stored at 4 °C until analysis.

### Determination of trace elements in dentine

A total of 25 elements were analyzed in a total of 150 samples of human coronal dentine. Trace elements were grouped as essential: Boron (B), Barium (Ba), Calcium (Ca), Cobalt (Co), Chrome (Cr), Copper (Cu), Iron (Fe), Potassium (K), Magnesium (Mg), Manganese (Mn), Sodium (Na), Nickel (Ni), Phosphorus (P), Sulfur (S), Selenium (Se), Strontium (Sr), Titanium (Ti), Vanadium (V), Zinc (Zn); and those that have no known biological role and/or are potentially toxic: Aluminum (Al), Arsenic (As), Cadmium (Cd), Lithium (Li), Lead (Pb), Stannum (Sn)^51^. The sensitive measurements for all trace elements analyzed were from 0.004 μg dm^−3^ to 13.543 μg dm^−3^.

Trace elements were determined by Inductively Coupled Plasma-Mass Spectrometry (ICP-MS, Agilent 7900 Series Instrument, Agilent Technologies, Dublin, Ireland), except for calcium (Ca) and phosphorus (P), which were determined by Inductively Coupled Plasma Atomic Emission Spectroscopy (ICP-OES, PerkinElmer, Waltham, MA, USA, Optima 8300). Calibration of ICP-MS was performed using multielement calibration standard solution, 1000 mg/L stock solution of each element (Agilent Technologies, USA, High-Purity Standards, USA and Merck, Germany) in 2% pure nitric acid. All of measurements had 3 replicas. Calibration of ICP-OES was carried out by means of a 1000 mg/L multielement calibration standard solution of the analyzed elements (High Purity Standards, Charleston, USA) and the working standards were prepared with acidified ultra-pure water (1% nitric acid, Panreac). The limit of detection for both teams was calculated as three times the standard deviation of the values of the blanks^11^. The working solutions were prepared by precisely measured dilution of the stock solutions with double distilled water for calibration.

### Statistical analysis

Demographic data and results were collected in a database (Microsoft Access 2.0; Microsoft Corporation, Seattle, WA) and the analysis was performed using SPSS 22.0 (SPSS software Inc., Chicago, IL) and the figures have been generated using GraphPad Prism 5 software. All results were expressed as mean ± SEM or as a percentage. The arithmetic mean (AM), standard error (SE), median, maximum (Max), minimum (Min), skewness and kurtosis values were estimated by descriptive statistical analysis. The nonparametric Mann-Whitney U test for two samples and the ANOVA Kruskal–Wallis test by ranks for multiple samples were run to compare mean values and mean values with different data groups, and Spearman’s rho correlation was used to measure the linear association between two variables. For continuous variables such as age, a receiver operation characteristic (ROC) curve was used to acquire a cut-off value for stratification. P-values below 0.05 were considered significant. The correlations between the elements present in teeth and age groups were assessed by regression analysis and principal component analysis (PCA). Kaiser–Meyer–Olkin’s measure of sampling adequacy (KMO) was used to assess the usefulness of a PCA in each age group. KMO ranges from 0 to 1 and should be well above 0.5 if variables are very interdependent and a PCA is useful. Kaiser-Meyer-Olkin’s measure of sampling adequacy (KMO) of a PCA in each age group were showed.

## Results

### Analysis of the concentration of trace elements in human tooth dentine

A total of 25 trace elements were analyzed in all the samples (n = 150) of coronary dentine (Supplementary Table 1), and all the toxic and essential elements mentioned above were detected. The trace element with the highest concentration was Ca (251937 ± 3868μg g-1) and the lowest was Cd (0.005 ± 0.003 μg g^−1^).

**Figure 1:**
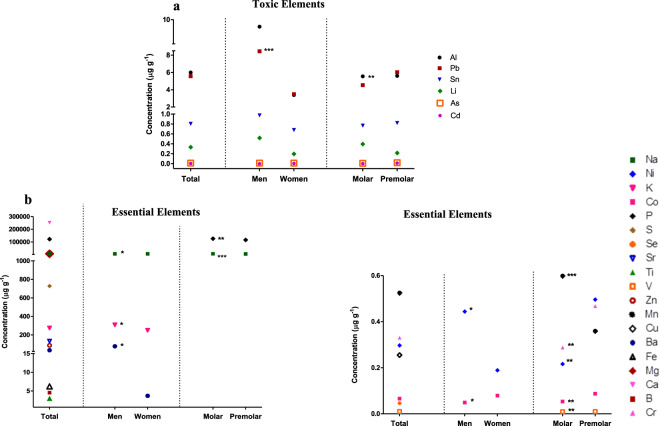
Analysis of the concentration of trace elements in tooth dentine. (**a**) The concentration of all toxic elements analyzed in the different study groups; (**b**) The concentration of essential elements with statistically significant differences between the different groups. Each point represents the mean concentration value of each trace element. Statistical analysis was performed using the Kruskal-Wallis test. P < 0.05 was taken to be statistically significant. *p < 0.05; **p < 0.01; ***p < 0.001. This figure has been generated using GraphPad Prism 5 software.

The concentrations of toxic elements were in the following order: Al > Pb > Sn > Li > As > Cd (Supplementary Table 1, Fig. 1a), and the essential elements followed the order of concentration: Ca > P > Mg > Na > S > K > Sr > Zn > Ba > Fe > B > Ti > Mn > Cr > Ni > Cu > Co > Se > V. The value of the concentration of each trace element is presented in Supplementary Table 1.

An analysis of the concentration of toxic elements according to sex and type of tooth was made (Fig. 1a). The concentration of Pb was significantly higher in men (8.452 ± 0.976 μg g^−1^) than in women (3.483 ± 0.831 μg g^−1^; P = 0.000). Similarly, Al concentrations in men (9.595 ± 4.734 μg g^−1^) were higher than in women’s teeth (3.394 ± 0.635 μg g^−1^) but the differences were not statistically significant. According to tooth type, similar concentrations of all toxic elements were detected in all cases.

The analysis of the concentration of essential elements according to the sex and type of tooth was also made (Fig. 1b). As regards sex, similar concentrations of trace most of the elements analyzed were observed, with only a statistically significant decrease in Na levels seen in men (6180 ± 161 μg g^−1^) compared with women (6481 ± 135 μg g^−1^; P = 0.032) and a statistically significant increase in K (309 ± 17 μg g^−1^), Co (0.049 ± 0.009 μg g^−1^) and Ni (0.444 ± 0.2 μg g^−1^) levels in men compared with the levels measured in women (Fig. 1b). The concentration of essential elements P (125868 ± 1953 μg g^−1^), Na (6739 ± 78.6 μg g^−1^), Mn (0.598 ± 0.043 μg g^−1^) were statistically higher in molars than in premolars, while Cr (0.287 ± 0.196 μg g^−1^), Ni (0.216 ± 0.116 μg g^−1^), Co (0.053 ± 0.007 μg g^−1^) and V (0.009 ± 0.0006 μg g^−1^) showed significantly lower levels in molars (Fig. 1b).

### Relationship between concentration of toxic elements in dentine and tooth age

**Figure 2:**
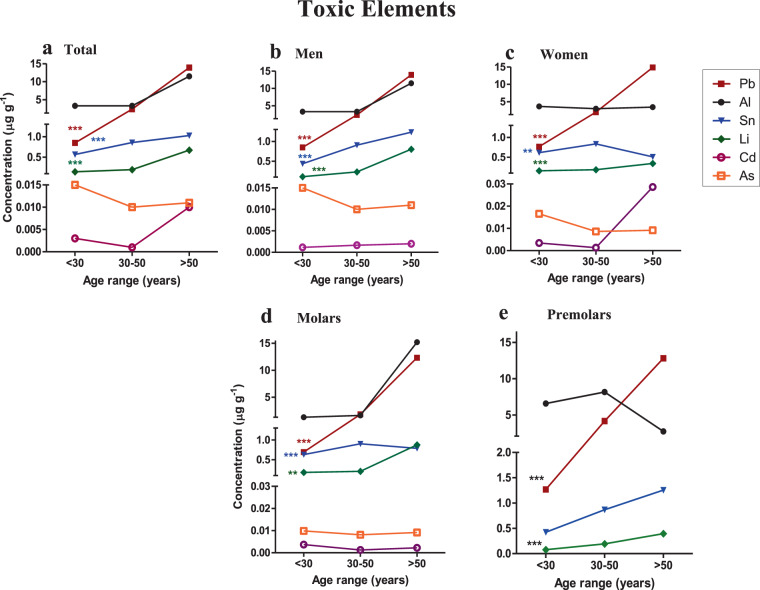
Concentration of toxic elements in dentine and their relationship with age. (**a**) Concentration of toxic elements in all the teeth. (**b,c**) Concentration of toxic elements in men and women’s teeth. (**d,e**) Concentration of toxic elements in the different types of teeth. Each point represents the mean concentration value of each trace element. Comparisons between the different age ranges (<30, 30–50, >50 years) were made using the Kruskal-Wallis test. P < 0.05 was taken as statistically significant. *p < 0.05; **p < 0.01; ***p < 0.001. This figure has been generated using GraphPad Prism 5 software.

The concentration of toxic elements in the three age ranges (<30, 30–50, >50 years) was analyzed in total teeth, according to sex and type of tooth (molar and premolar) (Fig. 2 and Supplementary Table 2).

Of the 6 toxic elements analyzed, only 3 elements (Li, Pb, Sn) were significantly related with age (P < 0.05) (Supplementary Table 1, Fig. 2a).

In total teeth, the toxic elements with the highest concentrations in both group I ( < 30 years) and group II (30–50 years) were Al > Pb > Sn > Li > As > Cd, while in group III ( > 50 years) Pb levels were higher than those of Al (Fig. 2a). A statistically significant increase of Pb, Sn and Li (P < 0.000) as the age range increased was observed in total teeth, men, women and molar groups (Fig. 2a–d). As regards Al, the concentrations remained constant in the <30 and 30–50 groups of teeth and increased in teeth >50 years but no significant differences were observed in any case. Similar results were found for Sn from men, women, and molars although those over 50 years did not show high levels in women or molars (Fig. 2a–e). The analysis of toxic elements in premolars showed a different distribution to the rest of the tooth groups analyzed but no significant relationship was found (Fig. 2e).

### Concentration of essential elements in tooth dentine and their relationship with age

**Figure 3:**
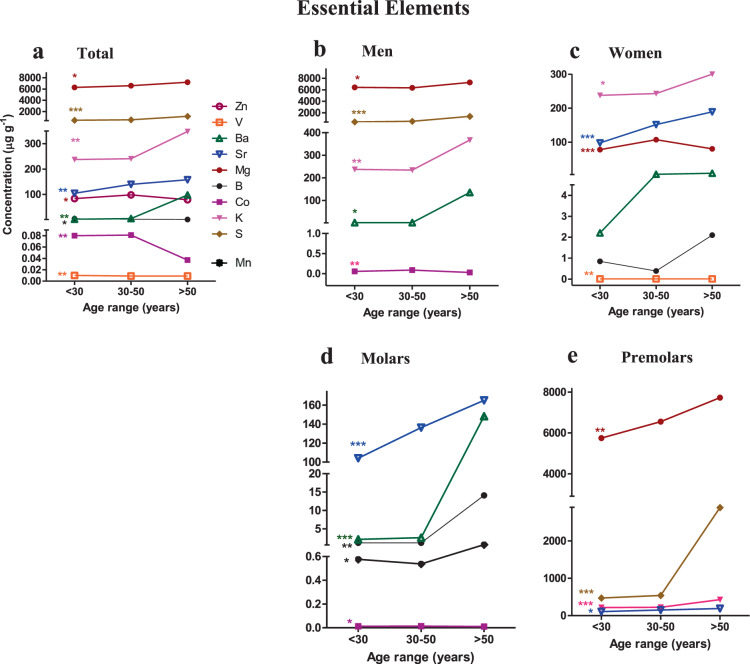
Concentration of essential elements in dentine and their relationship with age. (**a**) Concentration of essential elements in all the teeth (**b,c**). Concentration of essential elements in men and women teeth (**d,e**). Concentration of essential elements in the different types of teeth. Each point represents the mean concentration value of each trace element. Comparisons between the different age ranges (<30, 30–50, >50 years) were made by the Kruskal-Wallis test. P < 0.05 was statistically significant. *p < 0.05; **p < 0.01; ***p < 0.001. This figure has been generated using GraphPad Prism 5 software.

The concentration of essential elements in all age ranges (<30, 30–50, >50 years) were analyzed in total teeth, according to sex and type of tooth (Fig. 3 and Supplementary Table 3). Of the total of 19 essential elements analyzed, only 9 elements (B, Ba, Co, K, Mg, S, Sr, V, Zn) had a significant relation with age (P < 0.05). (Supplementary Table 1, Fig. 3a).

In total teeth, the highest concentrations of essential elements found showed the following order of concentration: Ca > P > Mg > Na > S > K > Sr > Zn > Ba.

As regards tooth age, small differences were found in group I (< 30 years) and group II (30–50 years) where the levels of Mg and Na were similar. In group III ( > 50 years), the concentration of Ba was higher than Zn.

In men, statistically significant differences were found for the following elements Mg, S, K, Co and Ba while for women it was K, Zn, Sr and V (Fig. 3c). The distribution of the concentration of these trace elements are presented in Fig. 3b,c. An analysis of concentrations according to tooth type identified statistically significant relationships with Ba, B, Co, Mn and Sr in molars (Fig. 3d) while in premolars S, Mg, K and Sr were significantly related (Fig. 3e).

### Analysis of the correlation between the different trace elements

Next, the correlation between different trace elements was considered (Table 2). While some trace elements showed significant correlations with each other. These correlations between elements suggest the possibility of agonistic and antagonistic absorption mechanisms in human teeth. However, correlation analysis between toxic elements pointed to significant positive correlations for Al-As/Pb, Pb-Li/Sn and Li-Sn.

**Table 2:** Correlation matrix for human teeth dentine giving values of Spearman’s Rho correlation coefficients for pairs of toxic and essential elements.

	Al	As	Cd	Pb	Li	Sn	B	Ba	Ca	Co	Cr	Cu	Fe	K	Mg	Mn	Na	Ni	P	S	Se	Sr	Ti	V	Zn
**Al**	1,000																								
**As**	0,216^**^	1,000																							
**Cd**	−0,281^**^	−0,104	1,000																						
**Pb**	0,280^**^	0,031	0,098	1,000																					
**Li**	0,136	−0,091	−0,119	0,387^**^	1,000																				
**Sn**	0,140	−0,100	0,060	0,459^**^	0,232^**^	1,000																			
**B**	0,558^**^	0,090	−0,112	0,359^**^	0,207^*^	0,262^**^	1,000																		
**Ba**	0,252^**^	0,082	−0,065	0,356^**^	0,097	0,218^**^	0,381^**^	1,000																	
**Ca**	−0,404^**^	−0,050	0,304^**^	−0,127	0,038	−0,012	−0,206^*^	0,077	1,000																
**Co**	0,526^**^	0,226^**^	−0,275^**^	0,019	−0,091	0,079	0,312^**^	0,117	−0,361^**^	1,000															
**Cr**	0,468^**^	0,283^**^	−0,250^**^	0,218^**^	0,136	0,073	0,188^*^	0,148	−0,247^**^	0,352^**^	1,000														
**Cu**	−0,406^**^	−0,278^**^	0,336^**^	0,009	−0,085	0,071	−0,244^**^	−0,126	0,241^**^	−0,447^**^	−0,292^**^	1,000													
**Fe**	0,545^**^	0,344^**^	−0,290^**^	0,135	0,103	0,107	0,315^**^	0,161^*^	−0,247^**^	0,675^**^	0,434^**^	−0,478^**^	1,000												
**K**	0,009	−0,012	−0,149	0,409^**^	0,410^**^	0,366^**^	0,048	0,243^**^	0,230^**^	−0,027	0,077	−0,105	0,248^**^	1,000											
**Mg**	−0,342^**^	−0,420^**^	0,059	0,169^*^	0,114	0,106	−0,056	0,026	0,213^**^	−0,412^**^	−0,390^**^	0,380^**^	−0,465^**^	0,216^**^	1,000										
**Mn**	−0,261^**^	−0,081	0,130	0,038	0,212^**^	0,139	−0,130	0,139	0,416^**^	−0,331^**^	−0,122	0,252^**^	−0,027	0,358^**^	0,213^**^	1,000									
**Na**	−0,313^**^	−0,365^**^	−0,116	−0,205^*^	0,254^**^	−0,019	−0,151	−0,023	0,410^**^	−0,358^**^	−0,302^**^	0,284^**^	−0,368^**^	0,185^*^	−0,412^**^	0,274^**^	1,000								
**Ni**	0,210^**^	0,054	−0,141	−0,015	−0,081	−0,025	0,012	−0,068	−0,289^**^	−0,001	0,180^*^	0,227^**^	−0,018	−0,370^**^	−0,390^**^	−0,162^*^	−0,064	1,000							
**P**	−0,350^**^	0,029	0,247^**^	−0,099	0,083	−0,030	−0,151	0,042	0,852^**^	−0,405^**^	−0,169^*^	0,054	−0,172^*^	0,229^**^	0,380^**^	0,432^**^	0,276^**^	−0,318^**^	1,000						
**S**	0,078	−0,165^*^	−0,147	0,285^**^	0,128	0,212^**^	−0,001	0,131	−0,218^**^	0,011	0,100	0,065	0,051	0,197^*^	−0,465^**^	−0,094	0,042	0,248^**^	−0,204^*^	1,000					
**Se**	−0,016	−0,131	−0,078	0,037	0,024	0,273^**^	0,033	0,074	−0,034	0,322^**^	−0,140	0,004	0,118	0,227^**^	0,216^**^	−0,092	0,136	−0,213^**^	−0,240^**^	−0,006	1,000				
**Sr**	−0,025	−0,070	0,136	0,332^**^	0,148	0,268^**^	−0,031	0,094	0,062	0,232^**^	−0,043	0,268^**^	0,134	0,312^**^	1,000	0,110	−0,068	−0,230^**^	−0,151	0,011	0,422^**^	1,000			
**Ti**	-0,531^**^	−0,303^**^	0,339^**^	−0,112	−0,033	−0,065	−0,315^**^	−0,131	0,348^**^	−0,611^**^	−0,472^**^	0,709^**^	−0,696^**^	−0,173^*^	0,047	0,185^*^	0,421^**^	0,214^**^	0,177^*^	0,000	−0,006	0,055	1,000		
**V**	0,101	−0,070	−0,072	−0,051	−0,060	0,068	0,033	0,028	−0,016	0,458^**^	−0,040	0,124	0,123	−0,006	0,521^**^	−0,076	0,026	0,109	−0,288^**^	0,040	0,456^**^	0,433^**^	0,035	1,000	
**Zn**	−0,218^**^	−0,312^**^	0,285^**^	0,148	0,029	0,274^**^	−0,140	0,097	0,109	−0,029	−0,346^**^	0,478^**^	−0,126	0,169^*^	−0,106	0,100	0,090	−0,142	−0,172^*^	0,157	0,437^**^	0,640^**^	0,342^**^	0,378^**^	1,000

The bold values represent significantly correlated parameter. *Correlation is significant at the 0.05 level (2-tailed). **Correlation is significant at the 0.01 level (2-tailed). Dark Grey represents the toxic elements. Light grey represents the essential elements.

**Figure 4:**
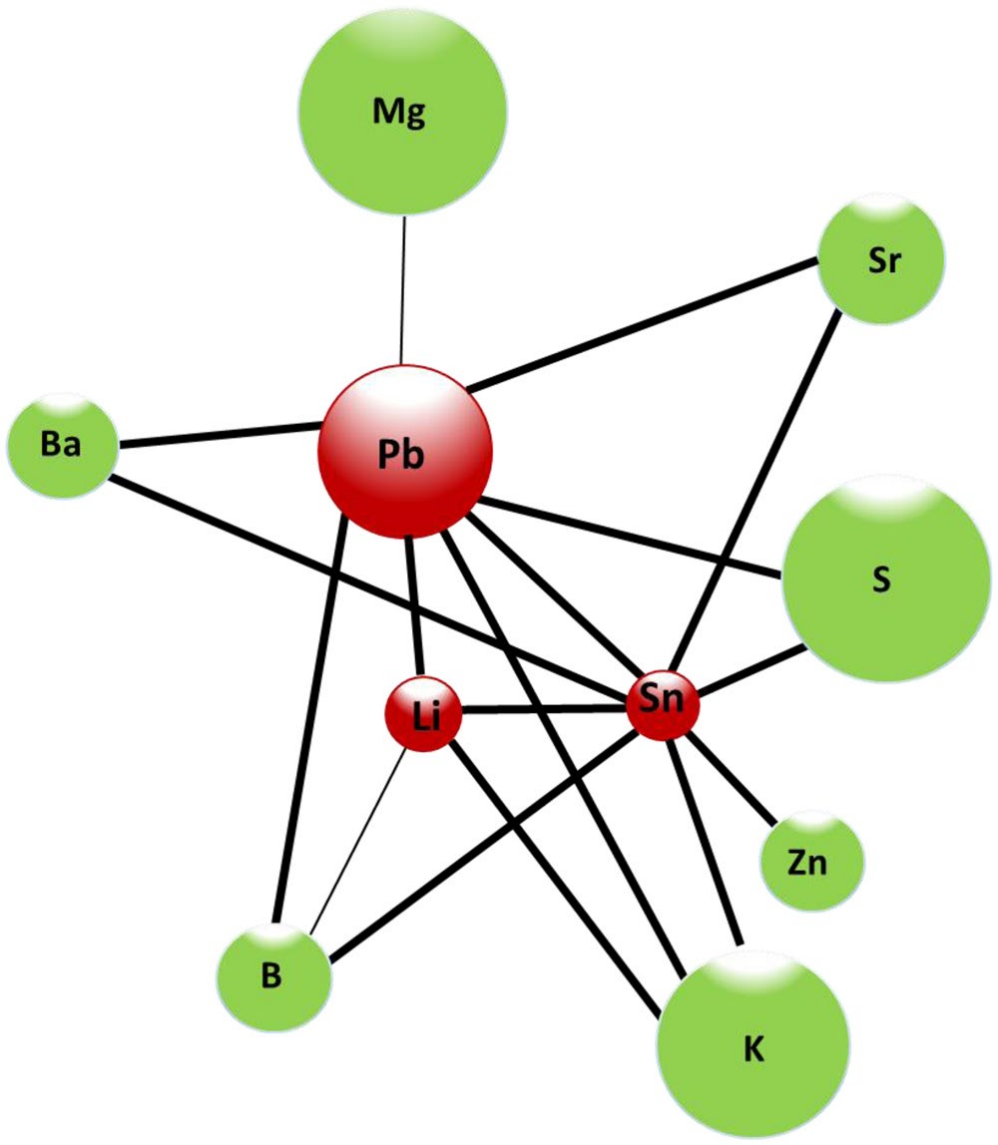
Representative diagram of the positive correlations between the toxic and essential elements that increase their concentration with age in human teeth dentine. Green nodes indicate essential elements. Red nodes indicate toxic elements. The thickness of the line indicates the degree of correlation. Fine line indicates a correlation is significant at the 0.05 level (2-tailed) and thick lines indicate that the correlation is significant at the 0.01 level (2-tailed). The size of the nodes represents the mean of the concentration of the element.

Significant positive correlation between different essential elements were also found; for example, Mg with V although the correlation was negative with S. The essential element K showed positive correlation with Mg, S, Sr and Zn, while Sr showed a significant positive correlation with V and Zn. Finally, a positive correlation was observed between essential and toxic elements including B with Al, Pb, Li, Sn. The rest of the correlations are presented in Table 2. In Fig. 4 shows the positive correlations between toxic and essential elements their concentration with age in human teeth dentine. The size of the nodes represents the mean of the concentration of the element. It should be noted that the toxic elements Pb and Sn are the elements that present the most positive correlations, reaching a total of 8 positive correlations with the rest of the elements that increase with age, both with essential and toxic elements. As shown in Fig. 4, Li has 4 positive correlations with K, Sn, B, Pb. The K have 3 positive correlations with Pb, Sn and Li. It should be noted that the S, Sr and Ba only show 2 positive correlations with the same elements Pb and Sn. Mg only has a single positive correlation with Pb.

### Correlation between the concentration of trace elements and age

The correlation coefficients between the concentrations of elements in dentine and age was analyzed (Table 3). Significant positive correlation was observed between trace element concentration (Li, Sn, Pb, B, Ba, K, Sr, S and Mg) and age.

**Table 3:** Significant positive correlation coefficients between trace elements in dentine and age.

	Toxic elements			Essential elements						
	Li	Sn	Pb	B	Ba	K	Sr	S	Zn	Mg
Correlation coefficient*	0.483	0.401	0.880	0.222	0.282	0.399	0.294	0.327	0.185	0.262
P value	0.000	0.000	0.000	0.006	0.000	0.000	0.000	0.000	0.023	0.001

*Values of Spearman’s Rho correlation coefficients.

The regression calculation was used to statistically describe the studied population. For dependencies of changes in age in the function of trace element concentration, Table 4 shows regression equations defining the age estimation by accumulation of trace elements in dentine.

**Table 4:** Statistical characteristics and trace elements concentration in the total teeth.

Trace elements	Regression equation	R^2^	P
**Toxic**			
Sn	y = 38.544 + 2.276*x	0.024	0.059
Pb	y = 29.944 + 1.872*x	0.639	**0.000**
Li	y = 39.682 + 2.073*x	0.025	0.055
**Essential**			
Sr	y = 32.734 + 0.058*x	0.073	**0.001**
Mg	y = 10.111 + 4.538*x	0.092	**0.000**
S	y = 39.156 + 1.673*x	0.022	0.071
K	y = 17.123 + 0.085*x	0.223	**0.000**
Zn	y = 41.472–0.013*x	0.001	0.729
Ba	y = 39.737 + 0.019*x	0.060	**0.003**
B	y = 39.449 + 0.212*x	0.059	**0.003**

R^2^, coefficient of determination. P, p-value. “y” represented the tooth age, “x” each trace element analyzed. *All teeth in the age range of 18 to 88 years are included. P-values marked in bold are statistically significant (P < 0.05).

The analysis of the total of teeth shows how all the toxic elements increased linearly with age, although only the increase in Pb was statistically significant (P = 0.000). Similarly, the rest of the essential elements were positively correlated with age, and these correlations were statistically significant in the case of Sr, Mg, K, Ba, B. The essential elements Zn showed a negative regression or decrease in concentration with increasing age.

**Figure 5:**
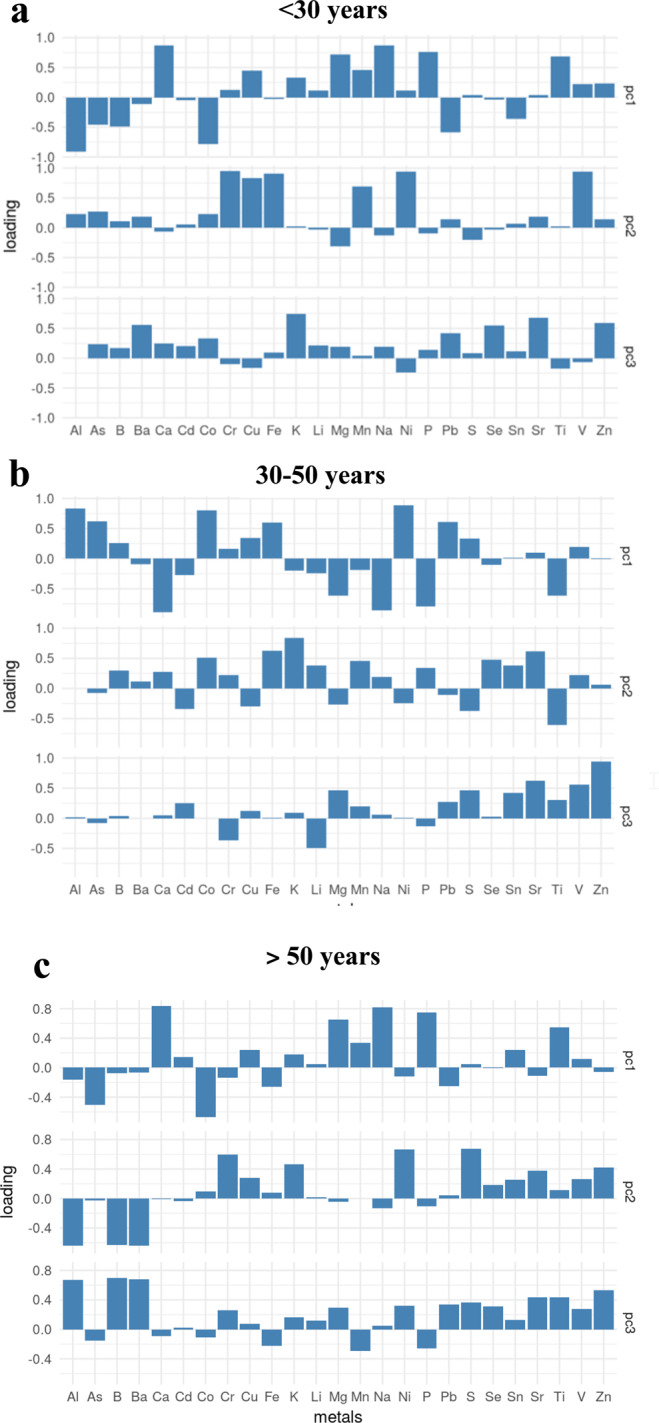
Results of principal component analysis in human coronal tooth dentine. (**a**) Analysis of trace elements in age group I (<30 years), (**b**) Analysis of trace elements in age group II (30–50 years), (**c**) Analysis of trace elements in age group III (>50 years). This figure has been generated using SPSS 22.0 (SPSS software Inc., Chicago, IL).

In addition, the PCA was used to identify whether the samples differed and what trace elements were responsible for any difference (Fig. 5). In group I ( < 30 years), KMO ( = 0.60) indicated the usefulness of a PCA, which explained 56% of the variation with three factors. Factor 1 was characterized by the high positive contribution of Ca, Mg, Na, P and Ti, while a negative contribution was observed in the case of Al, As, B, Co, Pb and Sn. As regards factor 2, Cr, Cu, Fe, Mn, Ni and V showed a strong positive contribution. Factor 3 was characterized by the high positive contribution of Ba, K, Se, Sr and Zn (Fig. 5a). In group II (30–50 years), KMO ( = 0.57) indicated the usefulness of a PCA, which explained 54% of the variation with three factors. Factor 1 was characterized by the high positive contribution of Al, As, Co, Fe, Ni, Pb and the high negative contribution of Ca, Mg, Na, P and Ti. In the case of factor 2, Co, Fe, K and Sr presented a strong positive contribution and Ti a strong negative contribution. Factor 3 was characterized by the high positive contribution of Sr, V and Zn (Fig. 5b). Finally, in group III (>50 years), KMO (=0.52) indicated the usefulness of a PCA, which explained 45% of the variation with three factors. Factor 1 of age group III (>50 years), was characterized by the positive contribution of Ca, Mg, Na, P and Ti, and a negative contribution on the part of As and Co. Factor 2 pointed to three high positive contributions on the part of Cr, Ni and S, while factor 3 was characterized by the high positive contribution of Al, B and Ba (Fig. 5c).

## Discussion

In this study, we have analyzed the concentration of twenty-five trace elements in the coronal dentine of healthy teeth according to sex to determine any relationship between the accumulation of these trace elements and age and, consequently, to be able to establish dentine as an appropriate long-term dental element for age studies in forensic investigation.

The trace elements incorporated in teeth enter the human body from different sources, such as food, water and air^11,52^. Trace elements have an important role both in general human health and dental health, since they participate in important biological processes, although in many cases the precise function is still unknown^53^. Previous studies indicated the importance of trace element analysis in dentine and permanent tooth enamel in order to provide chronological information on exposure, acting as a reliable bio-indicator of environmental pollution^11^.

The molars and premolars have been the dental pieces analyzed in this study because they are the most common to extract for orthodontic purposes^54,55^. Molars and premolars are the largest pieces, with the best easy handling for dentin extraction, and because they are the best protected teeth in the oral cavity. Both types of teeth emerge and mark the beginning of dental replacement. Furthermore, from the forensic point of view, in cases of destruction of the corpse by putrefaction or in situations of major catastrophes, they are usually the best preserved teeth^56^.

In our study cohort, we had an acceptable sample size for both types of teeth (109 molars and 41 premolars) and for reason and with the sole purpose of corroborating previous observations made by other authors in different populations^33,57^. Our results showed that with the exception of Zn in both types of teeth and B and Ba in premolars, the rest of the elements increase in each age group, reaching statistical significance in several of them.

Therefore, based on our results, both types of teeth could be used to estimate age. There is little and disparate information on the concentration of trace elements according to the type of tooth. Regarding lead accumulation, a study showed that lead accumulation depends on tooth type: the older the tooth, the higher its lead content^58^. On the other hand, other authors^59^ showed that tooth type is not a major factor affecting lead accumulation in adults. On the other hand, other authors analyzed 40 carious teeth and no found differences in the concentrations of trace elements between incisors, canines, molars^60^ and premolars, the Kumagai study also found no difference between tooth types^33^.

In our study, teeth were divided into three age groups and receptor operating characteristic curves (ROC) were used to find an optimal cut off value for each group with optimal sensitivity and specificity. It should be noted that giving a wide age range, about 20 years at least, is usual and recommended, especially in cases of deceased people and especially in adults. Since giving a minor age range can lead to incorrect dating^8^.

Different methods for estimating dental age using dental tissues and histological, radiographic and biochemical procedures are continuously being established^13–19^. Dental maturity plays an significant role in infant and adolescent age estimates^61^. The number and sequence of the outbreaked teeth will assess an individual’s age reasonably. Radiographical methods will focus further on the various stages of mineralization^62,63^ and further help in a more reliable age calculation. Mineralization of teeth provides a better approximation of chronological age than bone mineralization^64^ since the stage of mineralization in the teeth is less affected by variability in the individual’s nutritional and endocrine state. In this regard, the developmental stages of the teeth as given by Demirjian *et al*.^65^ are much in use for estimation of chronological age throughout the world^56^.

Several studies have analyzed how tooth age can be estimated based on aspartic acid racemization, morphological, histological and functional changes in teeth, dental imaging, changes in hardness and the modulus of elasticity of dentine^15,66–69^. Different authors suggest that reference values of trace elements in dentin can be useful for estimating age^33,70^, since there is no metabolism of the active element after the formation of dentin, and it is not affected by the oral environment, it is considered a reliable biological factor. But despite all this, dates on the trace elements found in tooth dentine is scarce^33,43^.

Dentin formation continues throughout the life of the tooth, and its formation results in a gradual but progressive reduction in the size of the pulp cavity. That the pulp supports the dentin and that age changes within the pulp are reflected in the dentin has been emphasized. Since the amount of trace elements in dentin may change with age, this is considered to be a reliable biological load index^33^. Electron microscopy demonstrates that these tissue spaces ultimately become mineralized, including all the constituent collagen structures and all the space (volume) between them. Currently there is uncertainty as to whether more mineral resides within collagen or outside it, and recently certain electron microscopic studies indicate far more mineral deposited between collagen than within it. Electron microscopy also shows that collagen structures differ considerably in the extracellular space (volume) they occupy, a result depending on the species and tissues examined as well as their age and maturation^71^.

Kumagai *et al*.^33^, in an analysis of the relation with age of 10 trace elements in dentine - boron (B), manganese (Mn), cobalt (Co),copper (Cu), zinc (Zn), rubidium (Rb), strontium (Sr), molybdenum (Mo), cadmium (Cd), and lead (Pb) - suggested that human dentine is an appropriate substance for relating sex and age, lending weight to the data obtained in our study.

Our data show that of the total of 25 analyzed elements, only twelve elements have a significant correlation with age, of which three are toxic or potentially toxic (Pb, Sn and Li) and nine are essential (Sr, Mg, S, K Zn, Ba, B, Co, V). In the case of the toxic or potentially toxic elements, all three (Li, Sn, Pb), have a significant and positive correlation with age, although the physiological function is not clear^72^. The analysis of lead (Pb) pointed to significant differences in terms of sex, with men presenting higher levels than women, corroborating the observations made by other authors^33^.

The analysis of the mean concentrations of Pb, Li and Sn found a significant increase in all three age ranges analyzed while the rest of the toxic elements not showed this trend. The positive correlation of Pb with age may be due to its affinity for collagen fibers and the fact that it accumulates in calcified tissue^73,74^, which has also been observed by other authors^11,33,75,76^. On the other hand, Pb is associated with environmental pollution processes^77,78^ and its accumulation can affect the concentrations of other essential elements such as Fe, Zn and Cu^75^. However, our data show that Pb accumulation significantly decreases the absorption of Na but not of the rest of the elements analyzed.

Regarding lithium (Li) and tin (Sn), Skalnaya *et al*.^79^ observed that both elements increase in concentration with age in women’s hair. Lithium (Li) is widely used as an effective treatment for mood disorders and is associated with increased risk of reduced urinary concentrating, hypothyroidism, hyperparathyroidism, and weight gain^80^. Tin (Sn) intoxication has been seen to alter the activities of some enzymes, affecting the metabolism of Zn, Cu, Fe and Ca and modifying the concentration of some other elements in tissues^11^.

In a study it was observed how the values of Li and Sn (1.30 V) were approximately equal to that of Li-Pb (1.25 V). Therefore, the transfer of electrons from Li to Sn and Pb would take place, respectively, leading to ionic bonds and preferred heterocoordination^81^. These effects are attributed to strong chemical interactions between the components. According to the model proposed by Marel^82^, they assume that the metals Li on the one hand and Pb or Sn on the other hand have conduction bands that partially overlap. As lithium is applied to Pb or Sn, some conduction electrons can flow from the Li band into the Pb or Sn band.

Furthermore, neutron diffraction measurements of liquid Li-Sn alloys provide evidence for appreciable ordering in the liquid, probably accompanied by charge transfer from Li to Sn. The structural properties of the Li-Sn alloy system closely resemble those of the Li-Pb system^83^.

As for the essential or potentially essential elements, the highest concentrations in dentine were observed for Ca, P, Mg and Na^84^. There were variations in Mg, Sr, B, Ba, K, S, Zn, Co and V with respect to the age of teeth, but only Mg, Sr, B, Ba, K, S and Zn concentrations showed significant positive correlations with age, increasing as age increases.

Kumagai *et al*.^33^ observed positive correlations between B, Co, Cu, Sr and Zn concentrations and age, corroborating our observations for B, Sr and Zn since the rest of the elements we analyzed were not analyzed in their study. Regarding cobalt and potassium, Derise *et al*.^43^ observed that the concentrations of both increased with age in the enamel; however our data do not agree with their observations for cobalt but do in the case of potassium.

Potassium (K) is involved in apatite biomineralization, along with numerous trace elements. In our study, it is the essential element that best explains the variation in concentration with age. Similar results were obtained in a study where different essential elements in dentin were analyzed and only K exhibited significant positive correlation with age^85^.

Magnesium is considered physiological calcium antagonist. This can serve as a significant regulator of cell functions at the cellular level. In healthy subjects the serum concentration is remarkably stable. Big average serum Mg concentrations are protected against various diseases^86^. Magnesium metabolism imbalances are normal and contribute to multiple pathological conditions^87^. Recent studies indicate that periodontitis may be a risk factor for cardiovascular diseases, which were also associated with deficiencies in Mg^88^. Interactions between and among different steps in the pathogenesis of periodontitis may explain the relationship between periodontal status and the Mg/Ca ratio.

Boron is an element commonly found in nature and the main source is food and drinking water, primarily through the use of fertilizers containing borate^89^. Boron has beneficial effects on lipid metabolism, obesity and thyroid metabolism and may be used as a cariostatic agent in dentistry^90^. Boron is believed to accumulate in bone several times greater than blood concentration and affects its calcium content^89^. Boron will deposit on teeth as calcium borate rather than calcium phosphate and change the properties of of teeth including its resistance to caries^91^. Our results show a negative correlation between B and Ca and a positive correlation between B-Li/Pb/Sn.

Strontium builds up in the bones and chronic or excessive exposure can cause metabolic dysfunctions and bone mineralization problems, lowering calcium in the bone and leading to hypocalcemia^11^. It has also been related with protection against tooth decay due to the exchange produced by calcium in hydroxyapatite^92^. It is an essential trace element in humans^93^. It is abundant in nature, it is present in all plants and animals, it is found in human tissues in relatively constant amounts, it is concentrated in the bones and teeth where it may have important functions such as bone and teeth hardening and dental caries prevention^94^. Many research has shown that having strontium in your diet improves the accumulation of dentin bone tissue in your teeth, and a lack of strontium in your diet causes your bones and teeth to become defectively mineralized. It has now been generally established that supplemented strontium as strontium ranelate decreases bone resorption and promotes osteoblast development and new bone formation in osteopor women^95^. It now appears that on osteoblastic cells there is also a particular receptor that responds to strontium alone and not to other minerals such as calcium or aluminum^93^. Our results show a strong correlation between Sr-Pb and Sr-Sn. In the case of strontium, other studies corroborate our observations, observing positive correlations between concentration and age^33^.

In the study carried out by Asaduzzaman *et al*.^11^ on 50 permanent teeth, it was observed that the concentration of Barium was higher in the older subjects, coinciding with our results. Barium is of minimal toxicity to humans in general and replaces calcium in dentine hydroxyapatite^24^ but high levels can cause acute intoxication^11^. Barium is found in a high percentage in bones and connective tissues and in small amounts in muscle, fat and skin^91^. Strontium in human bone has been found to increase regularly with age^96^. Some bone ligands are unlikely to unnecessarily discriminate between the small amounts of Group IIA elements. Large quantities of strontium are known it can cause rickets in laboratory animals, probably by displacing calcium^86^. Regarding the biological activities in mammals, there is some evidence that strontium could harden bones and teeth, making it considered an anticariogenic agent in men^86^. No demonstrable effects on a mammal’s growth were found. In fact, there is no evidence that barium has particular function for optimum bone formation, and low bone concentrations are likely to be inertial.

With regard to the concentrations of cobalt and zinc, no correlation with the three age groups was observed until the age of 50, when the concentration increases weakly and then decreases. Osteoblasts exposed to ions such as cobalt (Co) and chromium (Cr) undergo a dose-dependent proliferation reduction. Titanium (Ti) ions have been shown to be toxic at concentrations of 10 ppm or greater for 24 h. Additional past studies have shown that non-toxic metal ion concentrations affect the differentiation and function of osteoblast cells *in vitro*^86^.

## Conclusion

Our findings show an increase in the concentration of toxic (Pb, Li and Sn) and essential (B, Ba, K, Sr, S and Mg) elements in coronal dentin related to the age of the teeth, regardless of sex. The concentrations of Pb as a toxic element and K as an essential element are the elements that best explain the variability of age. According to our findings, both molars and premolars can be used to estimate age. Also when used in combination with other morphological or molecular examination of the unknown dental remains can the dental trace elemental composition be used as a forensic method.

It is necessary to increase studies on the concentrations of essential and toxic elements in dentin to increase the sample size and be able to reach normalized mean values of each element for each age, and thus be able to establish mathematical models that allow predicting the age. In view of our results, this will be our main objective in the future, since it will allow us to establish the age in forensic situations due to its long-term persistence.

## Supplementary information

Supplementary Tables.
