# Supplementary material for: Aging and trace elements in human coronal tooth dentine
Source: Sci Rep. 2020 Jun 19;10:9964. doi: 10.1038/s41598-020-66472-1 (PMC7305194; doi:10.1038/s41598-020-66472-1)
Supplement: Supplementary file 1 — Supplementary Tables. [file 41598_2020_66472_MOESM1_ESM.docx]

**Aging and trace elements in human coronal tooth dentine**

Ana C. Fernández-Escudero^1#^, Isabel Legaz^1#*^, Gemma Prieto-Bonete^1^, Manuel López-Nicolás^2^, Antonio Maurandi-López^3^, María D. Pérez-Cárceles^1^

#The contribution of these authors is equal and the order is arbitrary

| **Supplementary Table 1.** Concentration trace elements in total human coronal tooth dentine ordered from highest to lowest concentration. | | | | | | | | |
| --- | --- | --- | --- | --- | --- | --- | --- | --- |
|  | **Concentration**  **(μg g^-1^)*** | **Median** | **Min** | **Max** | **Skewness** | **Kurtosis** |  | **P*** |
| **Toxic elements** | |  |  |  |  |  |  |  |
| Al | 5.999 ± 2.029 | 0.381 | 0.005 | 229.86 | 7.926 | 65.789 |  | 0.314 |
| Pb | 5.570 ± 0.662 | 1.956 | 0.064 | 63.318 | 3.360 | 17.945 |  | **0.000** |
| Sn | 0.804 ± 0.105 | 0.456 | 0.035 | 9.571 | 4.145 | 20.533 |  | **0.000** |
| Li | 0.333 ± 0.117 | 0.185 | 0.000 | 17.731 | 12.037 | 146.530 |  | **0.000** |
| As | 0.012 ± 0.002 | 0.010 | 0.000 | 0.248 | 9.525 | 101.092 |  | 0.215 |
| Cd | 0.005 ± 0.003 | 0.001 | 0.001 | 0.385 | 11.345 | 133.171 |  | 0.943 |
| **Essential elements** | |  |  |  |  |  |  |  |
| Ca | 251937.1 ± 3868 | 260921.7 | 117433 | 411045.3 | -0.634 | 1.848 |  | 0.969 |
| P | 122143.3 ± 2092 | 126740.6 | 10950 | 199553.6 | -0.535 | 3.175 |  | 0.715 |
| Mg | 6668.6 ± 103.477 | 6526.13 | 3622.1 | 10950.1 | 0.184 | 0.331 |  | **0.004** |
| Na | 6354.8 ± 104.114 | 6565.9 | 2722 | 9954.380 | -0.815 | 0.558 |  | 0.682 |
| S | 727.83 ± 137.158 | 507.803 | 97.301 | 20587.0 | 11.305 | 133.5 |  | **0.000** |
| K | 274.82 ± 8.653 | 253.035 | 134.7 | 852.348 | 2.591 | 8.459 |  | **0.000** |
| Sr | 132.621 ± 7.274 | 121.198 | 0.649 | 477.697 | 0.591 | 0.778 |  | **0.004** |
| Zn | 86.680 ± 3.48 | 98.820 | 0.725 | 148.590 | -1.028 | -0.138 |  | **0.038** |
| Ba | 34.263 ± 20.4 | 2.363 | 0.090 | 2357.59 | 8.573 | 73.58 |  | **0.002** |
| Fe | 6.228 ± 0.854 | 2.315 | 0.000 | 88.997 | 5.223 | 35.706 |  | 0.602 |
| B | 4.354 ± 1.773 | 0.943 | 0.022 | 192.016 | 8.240 | 68.47 |  | **0.044** |
| Ti | 3.017 ± 0.158 | 2.393 | 0.165 | 6.345 | 0.036 | -1.696 |  | 0.367 |
| Mn | 0.525 ± 0.033 | 0.413 | 0.140 | 2.630 | 2.841 | 10.060 |  | 0.105 |
| Cr | 0.330 ± 0.142 | 0.010 | 0.000 | 19.690 | 9.719 | 104.50 |  | 0.199 |
| Ni | 0.297 ± 0.087 | 0.053 | 0.010 | 11.699 | 8.913 | 90.450 |  | 0.577 |
| Cu | 0.255 ± 0.050 | 0.087 | 0.002 | 6.164 | 7.098 | 62.175 |  | 0.142 |
| Co | 0.066 ± 0.007 | 0.010 | 0.000 | 0.252 | 0.814 | -1.104 |  | **0.011** |
| Se | 0.046 ± 0.007 | 0.01 | 0.001 | 0.674 | 3.604 | 16.934 |  | 0.614 |
| V | 0.010 ± 0.001 | 0.010 | 0.002 | 0.066 | 8.997 | 100.78 |  | **0.002** |
| * (AM ± SE). AM, Arithmetic mean; SE, standard error, Max, maximum, Min, minimum, P, p-value. P*, Comparisons between the different age ranges (<30, 30-50, ˃50 years) were made by the Kruskal-Wallis test. P-values marked in bold are statistically significant (P˂0.05). | | | | | | | | |

| **Supplementary Table 2.** Concentration of toxic elements in human coronal dentin in total cohort and by sex. | | | | | | | |
| --- | --- | --- | --- | --- | --- | --- | --- |
|  | **Age range** | **Aluminum (Al)** | **Arsenic (As)** | **Cadmium (Cd)** | **Lead (Pb)** | **Lithium (Li)** | **Tin (Sn)** |
| **Total** | 18-88 | 5.999 ± 2.029 | 0.012 ± 0.002 | 0.005 ± 0.003 | 5.570 ± 0.662 | 0.333 ± 0.117 | 0.804 ± 0.105 |
| **N=150** | <30 | 3.329 ± 0.766 | 0.015 ± 0.004 | 0.003 ± 0.002 | 0.848 ± 0.145 | 0.146 ± 0.015 | 0.568 ± 0.181 |
|  | 30-50 | 3.322 ± 0.862 | 0.010 ± 0.001 | 0.001 ± 0.000 | 2.377 ± 0.342 | 0.199 ± 0.018 | 0.857 ± 0.170 |
|  | >50 | 11.509 ± 6.064 | 0.011 ± 0.018 | 0.010 ± 0.008 | 13.900 ± 1.367 | 0.672 ± 0.356 | 1.026 ± 0.188 |
| ***P* values*** |  | 0.314 | 0.215 | 0.943 | **0.000** | **0.000** | **0.000** |
|  |  |  |  |  |  |  |  |
| **Men** | 18-88 | 9.596 ± 4.734 | 0.011 ± 0.002 | 0.002 ± 0.000 | 8.453 ± 0.977 | 0.520 ± 0.278 | 0.975 ± 0.183 |
| **N=63** | <30 | 2.374 ± 1.211 | 0.010 ± 0.001 | 0.001 ± 0.000 | 1.071 ± 0.364 | 0.110 ± 0.033 | 0.436 ± 0.181 |
|  | 30-50 | 4.121 ± 1.630 | 0.012 ± 0.003 | 0.002 ± 0.000 | 3.318 ± 0.782 | 0.231 ± 0.043 | 0.905 ± 0.503 |
|  | >50 | 14.725 ± 8.438 | 0.011 ± 0.003 | 0.002 ± 0.000 | 13.524 ± 1.139 | 0.803 ± 0.499 | 1.232± 0.255 |
| ***P* values*** |  | 0.140 | 0.443 | 0.874 | **0.000** | **0.001** | **0.001** |
|  |  |  |  |  |  |  |  |
| **Women** | 18-88 | 3.394 ± 0.635 | 0.012 ± 0.003 | 0.007 ± 0.005 | 3.483 ± 0.831 | 0.198 ± 0.014 | 0.680 ± 0.123 |
| **N=87** | <30 | 3.678 ± 0.950 | 0.017 ± 0.006 | 0.003 ± 0.002 | 0.766 ± 0.149 | 0.159 ± 0.016 | 0.616 ± 0.238 |
|  | 30-50 | 2.998 ± 1.027 | 0.009 ± 0.001 | 0.001 ± 0.000 | 1.995 ± 0.348 | 0.185 ± 0.018 | 0.837 ± 0.134 |
|  | >50 | 3.471 ± 1.629 | 0.009 ± 0.001 | 0.029 ± 0.028 | 14.840 ± 3.945 | 0.343 ± 0.048 | 0.510 ± 0.079 |
| ***P* values*** |  | 0.726 | 0.554 | 0.772 | **0.000** | **0.002** | **0.003** |
|  |  |  |  |  |  |  |  |
| **Molar** | 18-88 | 5.553 ± 2.954 | 0.010 ± 0.000 | 0.002 ± 0.009 | 4.537 ± 0.641 | 0.396 ± 0.773 | 0.769 ± 0.131 |
| **N=109** | <30 | 1.321 ± 0.484 | 0.010 ± 0.000 | 0.004 ± 0.003 | 0.693 ± 0.161 | 0.180 ± 0.018 | 0.630 ± 0.265 |
|  | 30-50 | 1.644 ± 0.479 | 0.008 ± 0.001 | 0.001 ± 0.000 | 1.849 ± 0.293 | 0.207 ± 0.022 | 0.904 ± 0.217 |
|  | >50 | 15.204 ± 9.803 | 0.009 ± 0.001 | 0.002 ± 0.000 | 12.324 ± 1.271 | 0.876 ± 0.582 | 0.788 ± 0.168 |
| ***P* values*** |  | 0.100 | 0.142 | 0.191 | **0.000** | **0.003** | **0.000** |
|  |  |  |  |  |  |  |  |
| **Premolar** | 18-88 | 5.602 ± 1.371 | 0.019 ± 0.007 | 0.013 ± 0.011 | 6.028 ± 1.057 | 0.217 ± 0.032 | 0.822 ± 0.182 |
| **N=41** | <30 | 6.586 ± 2.142 | 0.030 ± 0.170 | 0.001 ± 0.000 | 1.267 ± 0.375 | 0.077 ± 0.020 | 0.424 ± 0.172 |
|  | 30-50 | 8.174 ± 3.496 | 0.016 ± 0.006 | 0.002 ± 0.000 | 4.176 ± 1.179 | 0.193 ± 0.031 | 0.868 ± 0.245 |
|  | >50 | 2.740 ± 1.794 | 0.008 ± 0.001 | 0.033 ± 0.032 | 12.816 ± 1.385 | 0.396 ± 0.054 | 1.256 ± 0.426 |
| ***P* values*** |  | 0.614 | 0.447 | 0.578 | **0.000** | **0.000** | 0.109 |
| Elements concentration are expressed as μg g^-1^; (AM ± SE) AM. Arithmetic mean; SE. Standard error. *P values marked in bold are statistically significant (P ˂ 0.05). P. P-value obtained comparing different age groups. Comparisons were made by the Kruskal-Wallis test. | | | | | | | |

| **Supplementary Table 3.** Concentration of essential elements in human coronal dentin in total cohort and by sex. | | | | | | | |  |
| --- | --- | --- | --- | --- | --- | --- | --- | --- |
|  | **Age range** | **Boron (B)** | **Barium (Ba)** | **Potassium (K)** | **Strontium (Sr)** | **Sulfur (S)** | **Zinc (Zn)** | **Magnesium (Mg)** |
| **Total** | 18-88 | 4.354 ± 1.773 | 34.263 ± 20.467 | 274.820 ± 8.653 | 132.621 ± 7.274 | 727.833 ± 137.158 | 86.680 ± 3.488 | 6668.6 ± 103.477 |
| **N=150** | <30 | 2.076 ± 0.663 | 2.268 ± 0.145 | 237.928 ± 5.633 | 104.878 ± 7.445 | 479.542 ± 16.936 | 83.701 ± 4.644 | 6279.9 ± 151.451 |
|  | 30-50 | 1.426 ± 0.289 | 4.701 ± 2.134 | 240.816 ± 6.866 | 139.686 ± 9.866 | 549.762 ± 23.586 | 98.371 ± 5.158 | 6575.4 ± 173.778 |
|  | >50 | 9.646 ± 5.324 | 97.978 ± 62.057 | 348.213 ± 21.527 | 157.840 ± 17.771 | 1175.1 ± 414.342 | 79.348 ± 7.811 | 7198.5 ± 191.512 |
| ***P* values*** |  | **0.044** | **0.002** | **0.000** | **0.004** | **0.000** | **0.038** | **0.004** |
|  |  |  |  |  |  |  |  |  |
| **Men** | 18-88 | 7.650 ± 4.163 | 76.475 ± 48.425 | 309.082 ± 17.931 | 132.909 ± 12.196 | 986.681 ± 324.209 | 83.059 ± 6.254 | 6880.1 ± 166.024 |
| **N=63** | <30 | 1.516 ± 0.906 | 2.443 ± 0.372 | 237.828 ± 13.120 | 124.139 ± 12.250 | 433.339 ± 33.542 | 98.757 ± 4.838 | 6417.2 ± 256.220 |
|  | 30-50 | 1.225 ± 0.313 | 2.348 ± 0.358 | 234.374 ± 11.421 | 109.871 ± 24.358 | 538.506 ± 36.161 | 76.095 ± 14.927 | 6336.9 ± 368.049 |
|  | >50 | 12.665 ± 7.420 | 135.736 ± 86.398 | 367.369 ± 27.981 | 145.224 ± 19.306 | 1390.3 ± 577.753 | 78.919 ± 9.534 | 7280.3 ± 224.714 |
| ***P* values*** |  | 0.169 | **0.031** | **0.002** | 0.431 | **0.001** | 0.655 | **0.027** |
|  |  |  |  |  |  |  |  |  |
| **Women** | 18-88 | 1.968 ± 0.427 | 3.696 ± 1.115 | 250.011 ± 6.225 | 132.413 ± 8.968 | 540.398 ± 18.693 | 89.303 ± 3.966 | 6515.4 ± 130.175 |
| **N=87** | <30 | 2.281 ± 0.847 | 2.204 ± 0.147 | 237.964 ± 6.129 | 97.831 ± 8.958 | 496.446 ± 19.182 | 78.194 ± 5.889 | 6229.7 ± 185.569 |
|  | 30-50 | 1.508 ± 0.387 | 5.656 ± 2.995 | 243.433 ± 8.533 | 151.799 ± 9.198 | 554.335 ± 30.040 | 107.421 ± 2.987 | 6672.3 ± 194.743 |
|  | >50 | 2.099 ± 0.393 | 3.5848 ± 0.916 | 300.325 ± 24.952 | 189.378 ± 39.202 | 637.259 ± 70.281 | 80.422 ± 13.925 | 6993.8 ± 373.227 |
| ***P* values*** |  | 0.235 | 0.120 | **0.029** | **0.000** | 0.196 | **0.001** | 0.274 |
|  |  |  |  |  |  |  |  |  |
| **Molar** | 18-88 | 5.025 ± 2.607 | 45.678 ± 30.258 | 265.081 ± 6.627 | 133.045 ± 9.099 | 543.808 ± 18.353 | 87.406 ± 4.512 | 6762.7 ± 104.829 |
| **N=109** | <30 | 1.172 ± 0.410 | 2.127 ± 0.137 | 251.136 ± 5.990 | 104.096 ± 10.215 | 483.457 ± 21.326 | 83.978 ± 6.714 | 6571.9 ± 143.235 |
|  | 30-50 | 1.196 ± 0.296 | 2.604 ± 0.207 | 248.006 ± 7.768 | 136.363 ± 11.628 | 547.396 ± 25.781 | 97.601 ± 6.615 | 6662.1 ± 189.933 |
|  | >50 | 14.115 ± 8.629 | 148.206 ± 100.564 | 301.630 ± 17.644 | 164.989 ± 23.575 | 614.173 ± 45.066 | 80.078 ± 10.220 | 7112.1 ± 208.533 |
| ***P* values*** |  | **0.002** | **0.000** | 0.053 | **0.008** | 0.088 | 0.201 | 0.125 |
|  |  |  |  |  |  |  |  |  |
| **Premolar** | 18-88 | 2.845 ± 1.008 | 5.894 ± 2.864 | 292.962 ± 26.824 | 146.521 ± 14.052 | 1343.3 ± 597.227 | 91.621 ± 5.110 | 6634.9 ± 285.255 |
| **N=41** | <30 | 4.485 ± 2.352 | 2.805 ± 0.418 | 215.681 ± 10.401 | 107.363 ± 10.311 | 471.194 ± 35.953 | 84.502 ± 5.298 | 5745.5 ± 385.441 |
|  | 30-50 | 2.431 ± 0.948 | 14.685 ± 11.950 | 224.678 ± 12.158 | 149.243 ± 18.853 | 540.916 ± 75.796 | 102.232 ± 6.324 | 6552.5 ± 449.540 |
|  | >50 | 1.207 ± 0.310 | 3.637 ± 1.652 | 428.646 ± 57.570 | 190.392 ± 32.296 | 2895.8 ± 1639.7 | 92.852 ± 12.408 | 7727.4 ± 456.370 |
| ***P* values*** |  | 0.408 | 0.172 | **0.001** | **0.027** | **0.000** | 0.058 | **0.010** |
| Elements concentration are expressed as μg g^-1^; (AM ± SE) AM. Arithmetic mean; SE. Standard error. *P values marked in bold are statistically significant (P ˂ 0.05). P. P-value obtained comparing different age groups. Comparisons were made by the Kruskal-Wallis test. | | | | | | | | |

**DATABASE**

|  |  | **Li** | **B** | **Al** | **Ti** | **V** | **Cr** | **Mn** | **Fe** | **Co** | **Ni** | **Cu** | **Zn** | **As** | **Se** | **Sr** | **Cd** | **Sn** | **Ba** | **Pb** | **Ca** | **K** | **Mg** | **Na** | **P** | **S** |
| --- | --- | --- | --- | --- | --- | --- | --- | --- | --- | --- | --- | --- | --- | --- | --- | --- | --- | --- | --- | --- | --- | --- | --- | --- | --- | --- |
| **Teeth number** | **Age** | **µg/g ww** | **µg/g ww** | **µg/g ww** | **µg/g ww** | **µg/g ww** | **µg/g ww** | **µg/g ww** | **µg/g ww** | **µg/g ww** | **µg/g ww** | **µg/g ww** | **µg/g ww** | **µg/g ww** | **µg/g ww** | **µg/g ww** | **µg/g ww** | **µg/g ww** | **µg/g**  **ww** | **µg/g ww** | **µg/g ww** | **µg/g ww** | **µg/g ww** | **µg/g ww** | **µg/g ww** | **µg/g ww** |
| 1 | 24 | 0.219 | 11.171 | 27.180 | 2.612 | 0.010 | 6.198 | 0.323 | 12.565 | 0.252 | 0.946 | 0.010 | 87.440 | 0.248 | 0.010 | 163.617 | 0.001 | 2.460 | 6.270 | 4.997 | 153361.447 | 217.179 | 4101.803 | 4007.521 | 75224.210 | 432.121 |
| 2 | 51 | 0.190 | 4.985 | 29.291 | 1.036 | 0.010 | 0.349 | 0.289 | 14.227 | 0.229 | 0.901 | 0.010 | 79.741 | 0.096 | 0.010 | 122.826 | 0.001 | 0.942 | 7.114 | 9.683 | 150240.421 | 171.061 | 4687.244 | 3697.645 | 75745.847 | 713.001 |
| 3 | 48 | 0.133 | 3.377 | 15.371 | 0.696 | 0.010 | 0.196 | 0.149 | 7.076 | 0.186 | 0.782 | 0.010 | 84.745 | 0.010 | 0.010 | 97.629 | 0.001 | 0.532 | 2.410 | 6.806 | 140128.781 | 165.943 | 4537.272 | 3204.259 | 70044.717 | 654.096 |
| 4 | 48 | 0.149 | 2.585 | 15.965 | 1.675 | 0.010 | 0.202 | 0.261 | 10.330 | 0.224 | 0.957 | 6.164 | 102.575 | 0.053 | 0.010 | 112.694 | 0.001 | 0.229 | 2.347 | 7.817 | 158442.883 | 204.585 | 5144.403 | 3608.535 | 78785.833 | 667.740 |
| 5 | 48 | 0.133 | 2.133 | 10.085 | 0.882 | 0.010 | 0.112 | 0.172 | 10.495 | 0.225 | 0.988 | 0.010 | 75.099 | 0.010 | 0.010 | 113.134 | 0.001 | 0.520 | 2.048 | 6.507 | 154493.493 | 208.045 | 4666.758 | 3898.845 | 79754.988 | 698.119 |
| 6 | 19 | 0.075 | 1.029 | 8.596 | 0.901 | 0.010 | 0.089 | 0.173 | 9.093 | 0.192 | 0.823 | 0.010 | 60.115 | 0.029 | 0.010 | 55.555 | 0.001 | 0.080 | 1.702 | 2.857 | 135055.943 | 193.924 | 3622.147 | 3491.625 | 69322.747 | 420.207 |
| 7 | 30 | 0.116 | 0.810 | 8.848 | 0.809 | 0.010 | 0.105 | 0.215 | 9.657 | 0.205 | 0.833 | 0.010 | 69.459 | 0.035 | 0.010 | 89.918 | 0.001 | 0.077 | 2.977 | 1.373 | 149789.906 | 198.690 | 4586.584 | 3820.375 | 80596.724 | 486.131 |
| 8 | 23 | 0.132 | 33.398 | 8.621 | 0.969 | 0.010 | 0.329 | 0.197 | 9.277 | 0.197 | 0.899 | 0.010 | 58.990 | 0.037 | 0.010 | 98.191 | 0.001 | 0.123 | 1.886 | 2.079 | 147798.690 | 198.577 | 4555.047 | 3980.561 | 76859.139 | 435.528 |
| 9 | 23 | 0.137 | 1.247 | 12.474 | 0.840 | 0.010 | 0.156 | 0.239 | 9.725 | 0.223 | 0.972 | 0.010 | 73.731 | 0.010 | 0.010 | 107.209 | 0.001 | 0.080 | 1.606 | 1.788 | 165391.315 | 191.085 | 5347.660 | 4167.696 | 82522.467 | 432.880 |
| 10 | 60 | 0.533 | 2.251 | 22.016 | 0.990 | 0.010 | 0.289 | 0.201 | 11.010 | 0.202 | 0.880 | 0.010 | 91.631 | 0.010 | 0.010 | 198.296 | 0.001 | 0.080 | 5.379 | 21.992 | 154402.195 | 225.435 | 4814.911 | 3997.977 | 77043.885 | 1212.201 |
| 11 | 23 | 0.059 | 0.907 | 13.746 | 0.879 | 0.010 | 0.219 | 0.252 | 14.294 | 0.207 | 0.914 | 0.010 | 80.555 | 0.010 | 0.010 | 56.765 | 0.001 | 0.812 | 2.842 | 0.696 | 149181.173 | 192.860 | 5132.457 | 3808.985 | 75393.243 | 432.044 |
| 12 | 12 | 0.072 | 1.316 | 15.711 | 0.891 | 0.010 | 0.233 | 0.157 | 7.107 | 0.158 | 0.772 | 0.010 | 56.611 | 0.010 | 0.010 | 50.046 | 0.001 | 0.175 | 0.944 | 0.999 | 117433.259 | 134.753 | 4005.396 | 2722.124 | 58419.649 | 409.316 |
| 13 | 37 | 0.068 | 0.417 | 12.800 | 0.847 | 0.010 | 0.156 | 0.154 | 7.754 | 0.189 | 0.865 | 0.010 | 80.735 | 0.010 | 0.010 | 91.923 | 0.001 | 0.080 | 1.510 | 1.014 | 143704.223 | 161.030 | 4922.644 | 3771.413 | 71656.962 | 676.279 |
| 14 | 62 | 0.160 | 0.957 | 10.448 | 2.341 | 0.010 | 0.202 | 0.207 | 12.078 | 0.202 | 0.908 | 0.010 | 70.393 | 0.010 | 0.010 | 176.363 | 0.001 | 0.232 | 2.834 | 8.521 | 151922.487 | 236.493 | 5138.556 | 4172.901 | 78938.678 | 752.519 |
| 15 | 24 | 0.078 | 3.933 | 13.671 | 1.242 | 0.010 | 0.223 | 0.224 | 11.600 | 0.183 | 0.818 | 0.010 | 45.938 | 0.010 | 0.010 | 57.800 | 0.001 | 0.080 | 1.433 | 0.200 | 136993.645 | 152.996 | 3667.732 | 3564.760 | 68431.186 | 494.609 |
| 16 | 23 | 0.078 | 5.159 | 14.008 | 0.856 | 0.010 | 0.170 | 0.243 | 9.371 | 0.207 | 0.966 | 0.010 | 74.142 | 0.010 | 0.010 | 60.867 | 0.001 | 1.039 | 3.053 | 0.581 | 155865.744 | 201.639 | 5050.963 | 4110.575 | 79513.821 | 432.118 |
| 17 | 49 | 0.102 | 3.239 | 27.327 | 0.812 | 0.010 | 0.286 | 0.334 | 13.755 | 0.216 | 0.950 | 0.010 | 126.364 | 0.010 | 0.010 | 262.016 | 0.001 | 0.919 | 4.434 | 9.511 | 167387.587 | 212.592 | 5890.788 | 4298.296 | 83382.620 | 987.010 |
| 18 | 13 | 0.130 | 0.497 | 11.758 | 0.962 | 0.010 | 0.156 | 0.277 | 9.523 | 0.217 | 0.979 | 0.010 | 70.013 | 0.010 | 0.010 | 101.903 | 0.001 | 0.080 | 1.802 | 0.200 | 163678.521 | 200.066 | 4677.415 | 4240.489 | 82658.474 | 612.949 |
| 19 | 13 | 0.130 | 1.653 | 12.843 | 0.954 | 0.010 | 0.163 | 0.338 | 12.584 | 0.221 | 1.017 | 0.010 | 69.865 | 0.010 | 0.010 | 103.874 | 0.001 | 9.571 | 1.872 | 0.554 | 164511.581 | 202.684 | 4352.827 | 4445.503 | 82630.789 | 580.148 |
| 20 | 24 | 0.150 | 14.016 | 9.383 | 0.872 | 0.010 | 0.010 | 0.406 | 16.399 | 0.148 | 0.189 | 0.022 | 110.656 | 0.010 | 0.373 | 149.985 | 0.001 | 2.791 | 3.773 | 2.577 | 236398.903 | 266.965 | 7760.558 | 5917.728 | 118366.590 | 522.864 |
| 21 | 36 | 0.187 | 9.749 | 6.010 | 1.396 | 0.010 | 0.072 | 0.497 | 26.790 | 0.168 | 0.010 | 0.116 | 113.464 | 0.010 | 0.010 | 202.309 | 0.001 | 1.170 | 3.536 | 2.197 | 262595.399 | 306.934 | 6235.376 | 6572.996 | 125887.740 | 507.598 |
| 22 | 27 | 0.103 | 6.032 | 4.139 | 1.274 | 0.010 | 0.010 | 0.324 | 13.938 | 0.160 | 0.010 | 0.191 | 126.560 | 0.010 | 0.010 | 157.423 | 0.001 | 0.529 | 2.330 | 0.823 | 249286.012 | 301.937 | 7066.827 | 6220.655 | 125714.236 | 530.068 |
| 23 | 37 | 0.272 | 3.507 | 7.616 | 0.805 | 0.010 | 0.117 | 0.778 | 14.198 | 0.167 | 0.010 | 0.035 | 98.725 | 0.010 | 0.257 | 186.872 | 0.001 | 1.339 | 2.520 | 3.127 | 276807.014 | 298.610 | 5957.189 | 6961.153 | 135357.813 | 413.238 |
| 24 | 28 | 0.136 | 3.887 | 5.969 | 1.296 | 0.010 | 0.010 | 1.179 | 22.400 | 0.177 | 0.010 | 0.149 | 94.656 | 0.010 | 0.010 | 146.168 | 0.001 | 0.779 | 3.612 | 2.125 | 267656.834 | 295.542 | 6016.611 | 6353.492 | 130446.040 | 494.085 |
| 25 | 14 | 0.131 | 1.830 | 3.087 | 0.775 | 0.010 | 0.010 | 0.393 | 13.162 | 0.166 | 0.010 | 0.004 | 92.662 | 0.010 | 0.142 | 109.993 | 0.001 | 0.965 | 1.668 | 0.237 | 272434.290 | 279.084 | 6145.305 | 6550.640 | 135795.636 | 443.774 |
| 26 | 23 | 0.121 | 4.259 | 6.842 | 0.656 | 0.010 | 0.103 | 0.596 | 21.665 | 0.236 | 0.010 | 0.245 | 98.121 | 0.010 | 0.010 | 99.829 | 0.095 | 0.109 | 2.738 | 1.713 | 261665.583 | 296.858 | 6142.942 | 6370.745 | 127425.775 | 435.640 |
| 27 | 30 | 0.161 | 1.990 | 1.867 | 0.728 | 0.010 | 0.010 | 0.373 | 10.913 | 0.164 | 0.010 | 0.007 | 105.721 | 0.010 | 0.121 | 185.272 | 0.001 | 0.279 | 1.882 | 1.056 | 261693.975 | 295.282 | 6196.263 | 6385.340 | 129217.665 | 448.634 |
| 28 | 70 | 0.254 | 4.382 | 3.220 | 0.537 | 0.010 | 0.010 | 0.573 | 21.123 | 0.169 | 0.010 | 0.176 | 110.021 | 0.010 | 0.010 | 313.314 | 0.001 | 0.153 | 3.345 | 18.980 | 265119.980 | 269.015 | 7119.685 | 6884.974 | 129317.514 | 482.719 |
| 29 | 20 | 0.198 | 2.194 | 1.576 | 1.761 | 0.010 | 0.134 | 0.478 | 25.783 | 0.173 | 0.010 | 0.029 | 103.474 | 0.010 | 0.010 | 189.987 | 0.001 | 0.319 | 3.012 | 0.668 | 272944.392 | 289.984 | 5315.432 | 6805.624 | 132945.672 | 390.758 |
| 30 | 17 | 0.123 | 0.818 | 0.005 | 0.661 | 0.010 | 0.010 | 0.470 | 15.825 | 0.169 | 0.010 | 0.007 | 104.008 | 0.010 | 0.306 | 119.569 | 0.001 | 0.377 | 2.693 | 0.471 | 267744.793 | 320.641 | 5816.658 | 6576.411 | 133535.349 | 498.674 |
| 31 | 29 | 0.142 | 1.074 | 0.496 | 1.402 | 0.010 | 0.010 | 0.407 | 18.348 | 0.161 | 0.010 | 0.009 | 92.946 | 0.010 | 0.201 | 227.155 | 0.001 | 0.249 | 3.664 | 0.701 | 261480.286 | 272.804 | 6843.463 | 6426.623 | 126444.849 | 443.548 |
| 32 | 23 | 0.102 | 1.188 | 0.840 | 0.620 | 0.010 | 0.010 | 0.267 | 8.304 | 0.140 | 0.010 | 0.004 | 89.038 | 0.010 | 0.233 | 117.235 | 0.001 | 1.098 | 1.531 | 0.826 | 248159.993 | 245.834 | 6794.219 | 6174.865 | 121885.591 | 493.908 |
| 33 | 20 | 0.137 | 1.095 | 0.005 | 1.354 | 0.010 | 0.010 | 0.439 | 10.122 | 0.134 | 0.010 | 0.008 | 92.915 | 0.010 | 0.130 | 104.539 | 0.001 | 0.573 | 1.727 | 0.636 | 239581.612 | 266.491 | 7234.127 | 5813.803 | 117574.709 | 510.319 |
| 34 | 36 | 0.278 | 1.152 | 0.510 | 1.182 | 0.010 | 0.010 | 0.300 | 11.013 | 0.154 | 0.010 | 0.014 | 100.759 | 0.010 | 0.164 | 173.728 | 0.001 | 0.439 | 3.737 | 1.063 | 262987.094 | 270.716 | 5792.035 | 6580.743 | 127036.545 | 425.069 |
| 35 | 31 | 0.221 | 0.030 | 3.591 | 0.678 | 0.010 | 0.010 | 0.323 | 8.594 | 0.167 | 0.010 | 0.009 | 93.314 | 0.010 | 0.297 | 227.150 | 0.001 | 0.824 | 1.654 | 4.296 | 271177.258 | 262.990 | 6994.884 | 6853.936 | 132068.366 | 429.117 |
| 36 | 24 | 0.230 | 0.621 | 0.778 | 0.893 | 0.010 | 0.010 | 0.360 | 8.255 | 0.156 | 0.010 | 0.010 | 101.298 | 0.010 | 0.010 | 254.857 | 0.001 | 0.213 | 2.947 | 2.316 | 265823.655 | 292.089 | 6144.913 | 6695.931 | 129417.200 | 494.609 |
| 37 | 42 | 0.278 | 1.041 | 5.732 | 0.453 | 0.010 | 0.010 | 0.484 | 9.919 | 0.139 | 0.764 | 0.032 | 148.590 | 0.010 | 0.010 | 205.399 | 0.001 | 6.885 | 2.601 | 3.158 | 251526.745 | 291.995 | 6824.605 | 6214.524 | 123033.868 | 523.031 |
| 38 | 70 | 0.219 | 2.233 | 1.533 | 0.458 | 0.010 | 0.010 | 0.503 | 8.419 | 0.148 | 0.010 | 0.004 | 130.561 | 0.010 | 0.180 | 102.832 | 0.001 | 1.110 | 10.055 | 17.407 | 255316.263 | 579.571 | 7545.217 | 4882.037 | 124729.797 | 566.097 |
| 39 | 45 | 0.292 | 2.630 | 1.048 | 0.576 | 0.010 | 0.010 | 0.312 | 7.861 | 0.153 | 0.010 | 0.010 | 117.449 | 0.010 | 0.187 | 151.246 | 0.001 | 1.715 | 4.744 | 6.882 | 260959.064 | 264.639 | 6374.904 | 6327.052 | 127191.711 | 525.749 |
| 40 | 37 | 0.170 | 0.368 | 0.734 | 1.256 | 0.010 | 0.017 | 2.016 | 11.311 | 0.148 | 0.010 | 0.010 | 134.150 | 0.010 | 0.010 | 190.668 | 0.001 | 3.858 | 2.317 | 1.221 | 255295.570 | 293.223 | 5723.764 | 6154.034 | 125181.253 | 676.279 |
| 41 | 62 | 0.358 | 0.928 | 0.005 | 1.101 | 0.010 | 0.034 | 0.703 | 8.544 | 0.153 | 0.010 | 0.011 | 110.677 | 0.010 | 0.216 | 326.653 | 0.001 | 0.674 | 5.011 | 13.148 | 260926.352 | 315.238 | 6747.123 | 6380.186 | 128433.810 | 561.108 |
| 42 | 36 | 0.153 | 0.241 | 0.638 | 0.358 | 0.010 | 0.235 | 0.416 | 6.710 | 0.146 | 0.010 | 0.006 | 107.166 | 0.010 | 0.010 | 141.735 | 0.001 | 0.072 | 2.221 | 0.621 | 269180.283 | 265.350 | 6492.183 | 6588.164 | 129220.070 | 533.217 |
| 43 | 36 | 0.262 | 1.131 | 0.269 | 0.419 | 0.010 | 0.329 | 0.279 | 9.612 | 0.160 | 0.010 | 0.007 | 81.129 | 0.010 | 0.341 | 222.352 | 0.001 | 0.567 | 4.203 | 1.249 | 274464.215 | 291.959 | 4965.379 | 6919.282 | 134762.556 | 333.244 |
| 44 | 30 | 0.239 | 0.793 | 0.358 | 0.791 | 0.010 | 0.306 | 1.218 | 6.715 | 0.159 | 0.010 | 0.015 | 100.799 | 0.010 | 0.191 | 226.080 | 0.001 | 0.530 | 4.812 | 1.178 | 263293.175 | 277.602 | 6118.671 | 6437.338 | 128956.401 | 453.659 |
| 45 | 30 | 0.306 | 0.772 | 0.518 | 0.375 | 0.010 | 0.127 | 1.398 | 7.656 | 0.137 | 0.010 | 0.015 | 110.379 | 0.010 | 0.010 | 269.428 | 0.001 | 1.943 | 4.103 | 1.319 | 260419.636 | 376.121 | 5806.172 | 6444.304 | 128118.683 | 524.004 |
| 46 | 46 | 0.197 | 0.030 | 0.005 | 0.527 | 0.010 | 0.437 | 0.497 | 5.459 | 0.148 | 0.010 | 0.007 | 85.149 | 0.010 | 0.010 | 172.559 | 0.001 | 0.555 | 0.090 | 2.388 | 262300.620 | 273.607 | 7250.223 | 6668.097 | 129984.917 | 547.388 |
| 47 | 70 | 0.398 | 0.536 | 0.005 | 1.163 | 0.010 | 0.431 | 0.233 | 5.452 | 0.154 | 0.010 | 0.018 | 103.234 | 0.010 | 0.010 | 388.405 | 0.001 | 0.440 | 1.715 | 18.358 | 252242.398 | 492.951 | 6864.946 | 5277.486 | 121696.077 | 599.837 |
| 48 | 30 | 0.170 | 0.605 | 9.627 | 1.221 | 0.010 | 0.507 | 0.341 | 6.950 | 0.159 | 0.010 | 0.014 | 89.298 | 0.010 | 0.010 | 118.953 | 0.001 | 0.715 | 1.758 | 0.632 | 284216.124 | 305.489 | 5926.660 | 7043.142 | 137734.553 | 427.349 |
| 49 | 22 | 0.126 | 0.030 | 0.459 | 0.414 | 0.010 | 0.043 | 0.430 | 5.541 | 0.162 | 0.010 | 0.005 | 90.146 | 0.010 | 0.010 | 157.588 | 0.001 | 0.284 | 2.729 | 0.289 | 263354.464 | 315.196 | 6288.585 | 6552.368 | 129679.313 | 531.191 |
| 50 | 88 | 0.582 | 2.823 | 10.742 | 0.165 | 0.010 | 2.705 | 0.295 | 5.400 | 0.010 | 0.010 | 0.003 | 146.607 | 0.010 | 0.010 | 254.717 | 0.001 | 0.772 | 14.524 | 63.318 | 276120.406 | 511.788 | 6633.437 | 3562.368 | 127635.853 | 641.412 |
| 51 | 71 | 0.300 | 4.271 | 3.790 | 0.402 | 0.010 | 0.466 | 0.753 | 16.949 | 0.176 | 0.010 | 0.101 | 84.284 | 0.010 | 0.010 | 289.158 | 0.001 | 0.418 | 2.895 | 11.729 | 267698.961 | 387.613 | 6204.021 | 6045.637 | 131556.031 | 495.996 |
| 52 | 24 | 0.198 | 0.917 | 2.365 | 0.289 | 0.010 | 0.572 | 0.247 | 6.848 | 0.150 | 0.010 | 0.006 | 95.762 | 0.010 | 0.674 | 114.303 | 0.001 | 0.656 | 3.076 | 2.088 | 286383.357 | 300.557 | 6560.091 | 7098.603 | 140932.180 | 533.440 |
| 53 | 59 | 0.435 | 1.939 | 1.464 | 1.700 | 0.005 | 0.018 | 0.448 | 2.151 | 0.004 | 0.070 | 0.012 | 132.284 | 0.010 | 0.002 | 135.574 | 0.001 | 0.091 | 2.994 | 5.127 | 248361.381 | 204.407 | 7421.125 | 6422.386 | 119985.343 | 541.618 |
| 54 | 34 | 0.635 | 1.669 | 0.883 | 1.881 | 0.006 | 0.652 | 0.396 | 1.642 | 0.003 | 0.042 | 0.015 | 88.576 | 0.010 | 0.003 | 158.681 | 0.001 | 0.262 | 0.998 | 0.704 | 253148.888 | 222.035 | 6021.092 | 6671.772 | 124515.911 | 498.976 |
| 55 | 59 | 0.378 | 19.527 | 30.687 | 2.101 | 0.006 | 0.023 | 0.275 | 0.732 | 0.010 | 0.075 | 0.012 | 162.433 | 0.010 | 0.002 | 166.870 | 0.001 | 0.281 | 264.161 | 8.756 | 284920.900 | 257.323 | 9508.840 | 7489.890 | 147522.161 | 752.519 |
| 56 | 59 | 0.599 | 2.749 | 2.081 | 3.640 | 0.011 | 0.107 | 0.388 | 8.786 | 0.063 | 0.074 | 0.002 | 227.937 | 0.010 | 0.010 | 203.992 | 0.001 | 0.080 | 21.423 | 12.378 | 366383.372 | 377.501 | 10950.144 | 9954.380 | 199553.573 | 938.990 |
| 57 | 23 | 0.127 | 0.985 | 0.005 | 2.156 | 0.004 | 0.128 | 0.444 | 6.713 | 0.003 | 0.052 | 0.014 | 113.627 | 0.010 | 0.004 | 118.288 | 0.001 | 0.080 | 2.441 | 0.301 | 293572.818 | 260.165 | 7978.590 | 7546.786 | 147122.805 | 571.895 |
| 58 | 53 | 0.366 | 0.902 | 0.211 | 1.860 | 0.005 | 0.742 | 0.788 | 1.247 | 0.002 | 0.086 | 2.432 | 121.843 | 0.010 | 0.001 | 179.115 | 0.001 | 5.311 | 19.374 | 9.777 | 251915.049 | 239.570 | 5719.788 | 6458.359 | 131139.685 | 449.785 |
| 59 | 24 | 0.288 | 0.481 | 0.005 | 2.182 | 0.004 | 0.002 | 0.549 | 1.533 | 0.001 | 0.051 | 0.016 | 122.435 | 0.010 | 0.003 | 199.517 | 0.001 | 0.377 | 1.316 | 0.620 | 298694.590 | 256.785 | 6188.943 | 7444.470 | 152240.506 | 453.702 |
| 60 | 38 | 0.175 | 0.145 | 0.800 | 1.975 | 0.006 | 0.011 | 0.419 | 1.652 | 0.001 | 0.174 | 0.011 | 83.025 | 0.010 | 0.010 | 219.538 | 0.001 | 0.066 | 2.291 | 0.980 | 250245.009 | 222.664 | 5020.799 | 6475.791 | 131476.261 | 271.944 |
| 61 | 32 | 0.258 | 0.114 | 1.713 | 2.252 | 0.006 | 0.015 | 0.482 | 2.876 | 0.003 | 0.069 | 0.011 | 117.309 | 0.010 | 0.001 | 180.420 | 0.001 | 0.211 | 2.473 | 0.808 | 283982.028 | 275.826 | 5841.930 | 7105.846 | 147287.207 | 451.829 |
| 62 | 55 | 0.310 | 1.312 | 1.310 | 2.012 | 0.007 | 0.030 | 1.088 | 2.476 | 0.002 | 0.147 | 0.020 | 163.682 | 0.010 | 0.010 | 183.998 | 0.001 | 0.663 | 3.479 | 11.520 | 267565.382 | 309.788 | 6437.873 | 6759.125 | 139811.559 | 575.666 |
| 63 | 77 | 0.335 | 4.152 | 2.293 | 2.444 | 0.014 | 0.007 | 1.585 | 2.030 | 0.031 | 0.177 | 0.005 | 199.542 | 0.010 | 0.010 | 377.262 | 0.001 | 6.652 | 3.709 | 22.440 | 360369.048 | 684.812 | 7099.336 | 7527.390 | 197430.159 | 558.004 |
| 64 | 54 | 0.129 | 1.853 | 0.005 | 1.943 | 0.005 | 0.004 | 0.498 | 2.589 | 0.006 | 0.061 | 0.014 | 125.824 | 0.010 | 0.001 | 174.087 | 0.001 | 0.231 | 2.418 | 8.940 | 271306.566 | 240.355 | 6910.309 | 7198.873 | 143819.080 | 513.231 |
| 65 | 55 | 0.219 | 2.350 | 0.254 | 2.893 | 0.007 | 0.064 | 0.795 | 2.285 | 0.001 | 0.166 | 0.071 | 178.732 | 0.010 | 0.002 | 309.513 | 0.001 | 0.855 | 2.618 | 9.236 | 372119.816 | 434.542 | 9916.572 | 8252.264 | 190904.462 | 1219.760 |
| 66 | 64 | 0.074 | 0.975 | 0.005 | 1.344 | 0.006 | 0.470 | 0.227 | 0.423 | 0.010 | 0.042 | 0.008 | 170.957 | 0.010 | 0.001 | 194.180 | 0.001 | 1.189 | 9.137 | 22.489 | 180184.328 | 281.557 | 5683.550 | 3365.227 | 98293.168 | 494.540 |
| 67 | 87 | 0.334 | 1.726 | 5.674 | 1.801 | 0.004 | 0.005 | 0.258 | 1.535 | 0.001 | 0.035 | 0.011 | 115.158 | 0.010 | 0.001 | 142.672 | 0.001 | 0.760 | 2.425 | 13.374 | 250848.637 | 330.159 | 7109.665 | 6210.400 | 128222.554 | 557.646 |
| 68 | 61 | 0.169 | 0.672 | 0.670 | 1.874 | 0.008 | 3.163 | 0.411 | 0.912 | 0.010 | 0.040 | 0.013 | 111.200 | 0.010 | 0.002 | 273.343 | 0.001 | 0.385 | 5.141 | 16.332 | 260330.161 | 621.450 | 6430.573 | 5131.276 | 139544.073 | 737.834 |
| 69 | 26 | 0.181 | 1.097 | 0.799 | 1.823 | 0.008 | 0.014 | 0.521 | 1.826 | 0.001 | 0.132 | 0.028 | 111.308 | 0.010 | 0.001 | 114.897 | 0.001 | 0.154 | 2.162 | 0.339 | 278687.030 | 264.905 | 6413.767 | 7404.583 | 144729.576 | 528.585 |
| 70 | 26 | 0.121 | 0.771 | 0.220 | 1.703 | 0.004 | 0.029 | 0.427 | 0.718 | 0.002 | 0.050 | 0.018 | 114.355 | 0.010 | 0.001 | 82.724 | 0.001 | 0.139 | 1.836 | 0.590 | 232534.698 | 205.811 | 6679.615 | 6137.741 | 124869.414 | 501.484 |
| 71 | 53 | 0.452 | 3.151 | 1.324 | 1.975 | 0.018 | 0.031 | 2.630 | 1.586 | 0.010 | 0.077 | 0.022 | 110.753 | 0.010 | 0.010 | 232.131 | 0.001 | 0.795 | 4.550 | 4.967 | 257860.465 | 254.454 | 5175.680 | 6901.992 | 139742.497 | 460.306 |
| 72 | 77 | 0.622 | 1.308 | 4.768 | 1.777 | 0.006 | 0.011 | 0.400 | 2.425 | 0.010 | 0.026 | 0.008 | 114.219 | 0.010 | 0.003 | 357.012 | 0.001 | 0.255 | 1.859 | 10.291 | 234416.596 | 253.510 | 6626.478 | 5861.285 | 127486.071 | 510.295 |
| 73 | 21 | 0.086 | 2.190 | 1.813 | 2.112 | 0.005 | 0.018 | 0.387 | 0.526 | 0.010 | 0.045 | 0.013 | 101.561 | 0.010 | 0.010 | 108.871 | 0.001 | 2.180 | 1.420 | 0.721 | 268081.503 | 205.763 | 5858.411 | 7262.762 | 143767.336 | 420.399 |
| 74 | 34 | 0.410 | 1.225 | 0.359 | 1.911 | 0.005 | 0.019 | 0.473 | 6.245 | 0.036 | 0.050 | 0.023 | 87.935 | 0.001 | 0.002 | 187.442 | 0.001 | 0.324 | 1.320 | 0.823 | 245205.952 | 195.418 | 6723.047 | 6759.842 | 136576.866 | 496.457 |
| 75 | 56 | 0.089 | 0.030 | 0.389 | 1.796 | 0.007 | 0.015 | 0.898 | 1.756 | 0.010 | 0.038 | 0.012 | 119.356 | 0.010 | 0.010 | 211.307 | 0.001 | 0.587 | 2.415 | 13.482 | 235303.535 | 383.702 | 5475.184 | 4343.881 | 128915.238 | 680.847 |
| 76 | 21 | 0.427 | 0.328 | 0.005 | 1.844 | 0.003 | 0.005 | 0.375 | 1.223 | 0.010 | 0.028 | 0.010 | 101.519 | 0.010 | 0.001 | 87.330 | 0.001 | 0.046 | 0.914 | 0.151 | 246122.900 | 215.767 | 6352.642 | 6974.589 | 135383.260 | 480.435 |
| 77 | 25 | 0.316 | 0.030 | 0.005 | 1.962 | 0.003 | 0.010 | 0.343 | 1.616 | 0.010 | 0.034 | 0.008 | 106.808 | 0.010 | 0.010 | 84.549 | 0.001 | 0.043 | 0.888 | 0.162 | 244768.458 | 221.911 | 6157.589 | 6629.857 | 136434.358 | 486.115 |
| 78 | 75 | 0.487 | 183.682 | 229.868 | 2.743 | 0.008 | 0.142 | 0.444 | 1.340 | 0.010 | 0.039 | 0.009 | 134.967 | 0.010 | 0.010 | 354.396 | 0.001 | 1.334 | 2357.590 | 9.670 | 235330.246 | 227.170 | 7079.198 | 6558.803 | 131324.252 | 599.999 |
| 79 | 70 | 0.447 | 3.342 | 0.574 | 2.021 | 0.003 | 0.092 | 0.837 | 72.204 | 0.001 | 0.059 | 0.014 | 100.701 | 0.010 | 0.010 | 197.525 | 0.001 | 0.588 | 2.206 | 13.159 | 263167.088 | 365.311 | 6125.369 | 6742.304 | 143116.383 | 492.180 |
| 80 | 31 | 0.216 | 1.004 | 0.005 | 6.019 | 0.010 | 0.002 | 0.782 | 0.690 | 0.016 | 0.088 | 0.377 | 96.473 | 0.003 | 0.010 | 109.006 | 0.003 | 1.151 | 1.729 | 1.044 | 314764.366 | 224.997 | 6171.851 | 7017.413 | 152135.849 | 481.528 |
| 81 | 12 | 0.001 | 0.543 | 0.050 | 5.054 | 0.010 | 0.001 | 0.404 | 0.730 | 0.004 | 0.111 | 0.644 | 99.929 | 0.010 | 0.010 | 190.384 | 0.001 | 0.278 | 1.751 | 0.502 | 276022.477 | 205.815 | 7527.924 | 6205.590 | 137179.338 | 652.982 |
| 82 | 14 | 0.010 | 1.350 | 0.050 | 5.200 | 0.010 | 0.002 | 0.351 | 1.139 | 0.011 | 0.046 | 0.432 | 94.327 | 0.005 | 0.010 | 113.759 | 0.001 | 0.125 | 1.764 | 0.125 | 276558.099 | 213.494 | 7204.447 | 6344.747 | 136157.828 | 681.949 |
| 83 | 12 | 0.010 | 0.079 | 0.050 | 4.889 | 0.010 | 0.010 | 0.462 | 0.922 | 0.005 | 0.067 | 0.605 | 95.025 | 0.003 | 0.006 | 167.993 | 0.003 | 0.239 | 1.606 | 0.400 | 260086.508 | 207.951 | 7173.660 | 5864.620 | 127629.642 | 500.371 |
| 84 | 30 | 0.008 | 0.870 | 0.050 | 5.271 | 0.010 | 0.010 | 0.347 | 0.838 | 0.007 | 0.036 | 0.387 | 108.010 | 0.010 | 0.007 | 163.637 | 0.003 | 0.388 | 2.849 | 0.686 | 274653.277 | 195.762 | 6328.329 | 6484.571 | 135521.974 | 585.224 |
| 85 | 58 | 0.030 | 0.583 | 0.050 | 5.490 | 0.007 | 0.010 | 1.022 | 6.956 | 0.001 | 0.067 | 1.264 | 130.108 | 0.023 | 0.010 | 224.884 | 0.010 | 0.488 | 4.482 | 12.458 | 288955.737 | 453.980 | 7278.659 | 5241.978 | 141053.124 | 503.360 |
| 86 | 39 | 0.010 | 0.030 | 0.050 | 4.486 | 0.010 | 0.010 | 0.598 | 0.478 | 0.005 | 0.042 | 0.715 | 102.160 | 0.010 | 0.010 | 55.172 | 0.002 | 0.108 | 2.267 | 2.389 | 278280.103 | 169.914 | 6265.019 | 5824.887 | 132962.841 | 541.593 |
| 87 | 41 | 0.169 | 0.471 | 0.050 | 5.562 | 0.010 | 0.010 | 0.197 | 0.346 | 0.003 | 0.020 | 0.418 | 113.613 | 0.010 | 0.010 | 116.305 | 0.003 | 1.525 | 1.605 | 2.510 | 282683.855 | 189.855 | 7652.318 | 6342.275 | 137255.834 | 458.472 |
| 88 | 41 | 0.132 | 1.248 | 0.050 | 5.261 | 0.010 | 0.009 | 0.212 | 0.503 | 0.010 | 0.068 | 0.436 | 104.634 | 0.010 | 0.010 | 126.600 | 0.004 | 1.707 | 1.586 | 2.498 | 285813.596 | 195.921 | 7912.534 | 6480.840 | 141357.063 | 468.447 |
| 89 | 43 | 0.154 | 0.289 | 0.050 | 5.107 | 0.010 | 0.001 | 0.308 | 0.095 | 0.010 | 0.047 | 0.540 | 124.928 | 0.010 | 0.010 | 266.047 | 0.005 | 0.270 | 1.576 | 2.902 | 270537.928 | 280.103 | 7500.299 | 5353.235 | 135488.016 | 470.211 |
| 90 | 70 | 0.107 | 0.616 | 0.050 | 5.135 | 0.010 | 0.013 | 0.887 | 0.432 | 0.005 | 0.044 | 0.513 | 92.824 | 0.010 | 0.010 | 205.960 | 0.003 | 0.614 | 1.682 | 10.792 | 280454.658 | 213.729 | 5928.989 | 6421.747 | 134773.289 | 494.627 |
| 91 | 19 | 0.008 | 0.772 | 0.050 | 5.117 | 0.010 | 0.010 | 0.543 | 1.267 | 0.009 | 0.117 | 0.096 | 110.056 | 0.004 | 0.010 | 74.987 | 0.001 | 0.130 | 1.276 | 0.064 | 282672.055 | 208.843 | 6982.120 | 6403.264 | 137990.407 | 411.568 |
| 92 | 24 | 0.010 | 0.030 | 0.050 | 5.077 | 0.010 | 0.010 | 0.492 | 0.325 | 0.002 | 0.075 | 0.120 | 96.459 | 0.010 | 0.010 | 128.642 | 0.001 | 0.218 | 1.821 | 0.242 | 265947.071 | 196.798 | 7546.614 | 6432.461 | 125850.789 | 263.374 |
| 93 | 30 | 0.502 | 0.489 | 0.050 | 6.028 | 0.010 | 0.010 | 0.455 | 0.357 | 0.004 | 0.057 | 0.330 | 97.931 | 0.010 | 0.010 | 139.687 | 0.001 | 0.359 | 1.366 | 0.282 | 306182.202 | 203.249 | 5885.877 | 6841.943 | 145593.711 | 395.169 |
| 94 | 57 | 0.010 | 0.852 | 0.050 | 4.739 | 0.010 | 0.010 | 0.442 | 0.001 | 0.010 | 0.095 | 0.443 | 103.716 | 0.001 | 0.010 | 172.806 | 0.007 | 0.997 | 2.977 | 7.034 | 285980.386 | 198.709 | 7113.973 | 6699.216 | 134242.923 | 435.109 |
| 95 | 16 | 0.010 | 0.757 | 0.050 | 4.823 | 0.010 | 0.010 | 0.491 | 0.578 | 0.009 | 0.039 | 0.244 | 84.928 | 0.010 | 0.010 | 80.789 | 0.001 | 0.094 | 6.033 | 0.317 | 272897.162 | 208.930 | 6310.668 | 6165.418 | 132434.223 | 508.009 |
| 96 | 16 | 0.010 | 2.015 | 0.050 | 5.184 | 0.010 | 0.010 | 0.393 | 0.261 | 0.005 | 0.044 | 0.283 | 96.720 | 0.010 | 0.010 | 82.521 | 0.001 | 0.102 | 1.829 | 0.427 | 292526.148 | 228.959 | 6971.639 | 6625.468 | 142080.810 | 386.090 |
| 97 | 56 | 0.198 | 0.238 | 0.050 | 5.162 | 0.010 | 0.010 | 0.420 | 0.001 | 0.010 | 0.034 | 0.362 | 117.227 | 0.010 | 0.010 | 477.697 | 0.001 | 0.049 | 1.441 | 7.462 | 285924.120 | 196.774 | 7647.227 | 6260.801 | 136785.038 | 377.255 |
| 98 | 14 | 0.010 | 0.030 | 0.050 | 5.160 | 0.010 | 0.010 | 0.445 | 0.422 | 0.010 | 0.054 | 0.230 | 103.947 | 0.010 | 0.010 | 79.553 | 0.001 | 0.036 | 4.064 | 0.152 | 275827.792 | 219.898 | 6290.027 | 6283.149 | 133652.890 | 379.594 |
| 99 | 51 | 0.166 | 0.030 | 0.050 | 5.102 | 0.010 | 0.010 | 0.438 | 2.140 | 0.010 | 0.048 | 0.430 | 135.177 | 0.008 | 0.010 | 186.975 | 0.008 | 0.550 | 4.157 | 10.804 | 343391.916 | 188.513 | 7376.987 | 6092.578 | 164261.837 | 713.976 |
| 100 | 27 | 0.238 | 0.488 | 0.050 | 5.606 | 0.010 | 0.010 | 1.270 | 0.410 | 0.001 | 0.049 | 0.426 | 113.653 | 0.010 | 0.010 | 163.785 | 0.001 | 0.101 | 2.133 | 0.387 | 238644.765 | 218.976 | 6434.409 | 6734.225 | 116741.973 | 404.653 |
| 101 | 24 | 0.010 | 0.030 | 0.050 | 5.422 | 0.010 | 0.010 | 0.312 | 0.485 | 0.010 | 0.097 | 0.096 | 122.080 | 0.010 | 0.010 | 114.861 | 0.001 | 0.126 | 3.052 | 0.536 | 411045.320 | 197.250 | 6358.759 | 6105.022 | 199232.145 | 97.301 |
| 102 | 54 | 0.417 | 1.023 | 0.050 | 4.607 | 0.010 | 0.010 | 0.481 | 2.345 | 0.006 | 0.036 | 0.215 | 132.062 | 0.004 | 0.010 | 211.989 | 0.002 | 0.673 | 1.238 | 8.125 | 213531.626 | 263.757 | 6224.557 | 5585.143 | 102578.248 | 400.977 |
| 103 | 54 | 0.701 | 0.317 | 0.050 | 4.253 | 0.010 | 0.010 | 0.776 | 3.806 | 0.010 | 0.101 | 0.275 | 146.114 | 0.010 | 0.010 | 170.507 | 0.009 | 0.711 | 1.805 | 5.929 | 279970.323 | 251.091 | 5821.423 | 6087.118 | 133332.968 | 687.070 |
| 104 | 27 | 0.412 | 0.777 | 0.050 | 5.023 | 0.010 | 0.010 | 1.522 | 0.714 | 0.005 | 0.027 | 0.240 | 112.744 | 0.010 | 0.010 | 149.293 | 0.006 | 0.254 | 2.357 | 0.396 | 289346.408 | 239.894 | 5970.648 | 6705.442 | 140182.850 | 275.846 |
| 105 | 58 | 0.110 | 0.912 | 0.050 | 6.345 | 0.008 | 0.010 | 0.728 | 0.938 | 0.005 | 0.036 | 0.319 | 117.576 | 0.010 | 0.010 | 309.761 | 0.005 | 0.731 | 4.322 | 10.237 | 321207.105 | 349.103 | 6187.061 | 6964.840 | 153894.280 | 390.728 |
| 106 | 25 | 0.119 | 0.030 | 0.050 | 5.082 | 0.066 | 19.690 | 2.595 | 88.997 | 0.049 | 11.699 | 2.940 | 86.150 | 0.023 | 0.010 | 104.825 | 0.001 | 0.035 | 2.159 | 0.064 | 303710.875 | 242.704 | 5628.904 | 7451.073 | 142890.576 | 357.854 |
| 107 | 47 | 0.010 | 0.074 | 0.050 | 5.219 | 0.002 | 0.022 | 0.365 | 2.424 | 0.010 | 0.025 | 0.406 | 135.792 | 0.010 | 0.076 | 94.764 | 0.005 | 1.083 | 4.498 | 7.638 | 284424.523 | 236.335 | 7751.036 | 5637.418 | 135772.777 | 499.197 |
| 108 | 26 | 0.128 | 0.030 | 0.050 | 4.726 | 0.003 | 0.010 | 0.888 | 1.012 | 0.010 | 0.013 | 0.183 | 111.666 | 0.010 | 0.010 | 151.553 | 0.001 | 0.117 | 1.934 | 0.412 | 289218.118 | 216.249 | 6378.009 | 6390.910 | 139098.171 | 385.037 |
| 109 | 21 | 0.272 | 0.030 | 0.299 | 5.012 | 0.010 | 0.010 | 0.896 | 1.193 | 0.010 | 0.090 | 0.115 | 94.268 | 0.008 | 0.001 | 137.187 | 0.001 | 0.108 | 2.799 | 0.175 | 261130.526 | 244.326 | 7417.458 | 7628.054 | 129837.347 | 690.157 |
| 110 | 30 | 0.245 | 0.030 | 0.372 | 5.333 | 0.010 | 0.805 | 0.671 | 0.748 | 0.010 | 0.064 | 0.431 | 90.831 | 0.002 | 0.010 | 172.945 | 0.001 | 0.298 | 1.514 | 1.458 | 278442.843 | 271.588 | 7878.113 | 8467.619 | 131880.353 | 492.376 |
| 111 | 30 | 0.338 | 8.432 | 11.734 | 4.981 | 0.010 | 0.013 | 0.425 | 0.470 | 0.010 | 0.071 | 0.275 | 92.932 | 0.001 | 0.061 | 162.219 | 0.001 | 0.475 | 98.277 | 1.359 | 271370.337 | 259.550 | 6980.680 | 7965.749 | 126061.103 | 242.283 |
| 112 | 24 | 0.237 | 0.030 | 0.215 | 5.034 | 0.010 | 0.010 | 0.380 | 1.962 | 0.010 | 0.070 | 0.217 | 126.473 | 0.002 | 0.023 | 97.802 | 0.001 | 0.097 | 1.950 | 0.317 | 258577.055 | 253.744 | 7600.054 | 8196.644 | 121355.209 | 754.798 |
| 113 | 22 | 0.107 | 0.030 | 0.050 | 4.795 | 0.010 | 0.010 | 0.315 | 0.422 | 0.010 | 0.065 | 0.179 | 91.964 | 0.010 | 0.029 | 82.244 | 0.001 | 0.251 | 2.414 | 0.152 | 255217.645 | 259.530 | 7936.621 | 7986.056 | 119698.302 | 699.869 |
| 114 | 77 | 0.002 | 0.030 | 0.304 | 4.001 | 0.010 | 0.010 | 0.140 | 0.660 | 0.010 | 0.052 | 0.493 | 112.192 | 0.007 | 0.074 | 202.548 | 0.001 | 0.641 | 1.493 | 10.143 | 221756.945 | 281.430 | 8620.593 | 7886.223 | 105071.520 | 598.743 |
| 115 | 22 | 0.225 | 0.030 | 0.050 | 4.968 | 0.010 | 0.010 | 0.306 | 0.597 | 0.010 | 0.055 | 0.086 | 103.910 | 0.010 | 0.029 | 88.438 | 0.001 | 0.194 | 1.690 | 0.291 | 248919.477 | 260.749 | 8289.290 | 7744.083 | 119456.194 | 508.317 |
| 116 | 61 | 0.447 | 0.754 | 0.160 | 4.504 | 0.010 | 0.011 | 0.259 | 1.741 | 0.010 | 0.225 | 1.114 | 114.068 | 0.011 | 0.010 | 207.967 | 0.001 | 2.841 | 1.270 | 15.064 | 242900.462 | 852.348 | 8099.497 | 5623.871 | 117318.030 | 1101.478 |
| 117 | 61 | 0.484 | 0.593 | 0.561 | 4.346 | 0.010 | 4.535 | 0.429 | 14.405 | 0.010 | 5.112 | 0.533 | 128.650 | 0.003 | 0.074 | 213.525 | 0.001 | 2.873 | 1.212 | 12.757 | 260235.825 | 609.654 | 7971.951 | 6619.031 | 125580.618 | 20587.066 |
| 118 | 61 | 0.557 | 0.030 | 1.162 | 5.050 | 0.010 | 0.655 | 0.757 | 6.469 | 0.010 | 0.963 | 0.611 | 104.087 | 0.011 | 0.010 | 234.841 | 0.001 | 4.765 | 1.369 | 6.632 | 276694.751 | 545.072 | 5917.588 | 6757.216 | 133382.788 | 3954.346 |
| 119 | 61 | 0.260 | 0.030 | 0.164 | 4.079 | 0.010 | 0.537 | 0.279 | 2.367 | 0.010 | 0.683 | 0.560 | 113.288 | 0.006 | 0.072 | 198.823 | 0.001 | 1.500 | 1.246 | 14.314 | 244850.499 | 632.093 | 7705.719 | 5960.064 | 115706.604 | 3174.826 |
| 120 | 56 | 0.186 | 0.601 | 0.992 | 4.848 | 0.010 | 0.110 | 0.774 | 3.521 | 0.010 | 0.186 | 0.320 | 132.428 | 0.010 | 0.010 | 170.737 | 0.001 | 0.808 | 4.178 | 10.759 | 248249.084 | 259.029 | 8180.740 | 7688.734 | 115667.328 | 1177.624 |
| 121 | 60 | 0.180 | 0.164 | 0.050 | 4.210 | 0.010 | 0.038 | 0.650 | 0.579 | 0.010 | 0.085 | 0.494 | 118.140 | 0.004 | 0.010 | 151.146 | 0.001 | 0.811 | 2.759 | 6.270 | 262494.891 | 439.858 | 9213.998 | 7358.051 | 121230.005 | 1213.880 |
| 122 | 37 | 0.130 | 0.030 | 0.099 | 4.494 | 0.010 | 0.001 | 0.267 | 3.316 | 0.010 | 0.072 | 0.429 | 148.164 | 0.002 | 0.010 | 122.829 | 0.001 | 0.913 | 1.636 | 1.203 | 246091.868 | 248.994 | 6626.097 | 7308.314 | 112354.320 | 838.538 |
| 123 | 22 | 0.130 | 1.468 | 0.050 | 4.319 | 0.010 | 0.010 | 0.376 | 0.708 | 0.010 | 0.075 | 0.103 | 110.818 | 0.010 | 0.010 | 84.956 | 0.001 | 0.340 | 1.711 | 0.296 | 250721.251 | 227.780 | 7636.004 | 7358.618 | 115245.293 | 712.856 |
| 124 | 77 | 0.341 | 0.652 | 0.280 | 5.591 | 0.010 | 0.010 | 1.768 | 1.314 | 0.010 | 0.083 | 0.306 | 124.823 | 0.010 | 0.065 | 267.008 | 0.001 | 0.920 | 1.804 | 8.487 | 251239.342 | 289.962 | 8303.213 | 7470.973 | 113464.973 | 779.005 |
| 125 | 30 | 0.071 | 0.140 | 0.050 | 4.891 | 0.010 | 0.010 | 0.353 | 0.626 | 0.010 | 0.038 | 0.099 | 109.548 | 0.002 | 0.089 | 93.956 | 0.001 | 0.279 | 2.174 | 0.466 | 256369.715 | 229.430 | 7898.698 | 7883.820 | 113439.285 | 638.588 |
| 126 | 72 | 0.302 | 1.157 | 0.327 | 5.404 | 0.010 | 0.010 | 0.405 | 0.568 | 0.010 | 0.069 | 0.161 | 110.285 | 0.006 | 0.184 | 301.770 | 0.001 | 0.613 | 2.677 | 13.748 | 247773.275 | 367.686 | 9234.218 | 7500.049 | 111013.652 | 822.466 |
| 127 | 72 | 0.154 | 2.004 | 0.124 | 5.240 | 0.010 | 0.010 | 0.701 | 0.472 | 0.010 | 0.044 | 0.149 | 113.295 | 0.010 | 0.298 | 246.801 | 0.001 | 0.656 | 2.622 | 14.060 | 250367.577 | 300.316 | 7872.667 | 6387.361 | 111604.890 | 653.374 |
| 128 | 68 | 0.366 | 2.035 | 1.162 | 5.098 | 0.010 | 0.010 | 0.329 | 1.775 | 0.010 | 0.050 | 0.237 | 115.566 | 0.010 | 0.010 | 249.052 | 0.001 | 0.361 | 15.398 | 16.533 | 273177.710 | 267.311 | 7783.789 | 7878.011 | 124195.658 | 457.411 |
| 129 | 30 | 0.242 | 1.472 | 0.008 | 5.060 | 0.010 | 0.010 | 0.190 | 0.691 | 0.010 | 0.053 | 0.046 | 121.349 | 0.001 | 0.046 | 88.199 | 0.001 | 0.742 | 3.845 | 0.512 | 260917.147 | 229.998 | 8664.434 | 7628.214 | 117269.639 | 809.051 |
| 130 | 30 | 0.239 | 0.656 | 0.050 | 5.371 | 0.010 | 0.010 | 0.266 | 0.341 | 0.010 | 0.052 | 0.148 | 109.009 | 0.010 | 0.215 | 100.325 | 0.001 | 0.415 | 2.664 | 0.464 | 261213.734 | 221.526 | 7813.238 | 8062.850 | 118097.013 | 817.299 |
| 131 | 22 | 0.082 | 0.030 | 0.050 | 4.913 | 0.010 | 0.010 | 0.302 | 0.282 | 0.010 | 0.038 | 0.121 | 93.631 | 0.010 | 0.010 | 96.428 | 0.001 | 0.130 | 1.720 | 0.276 | 262677.575 | 218.333 | 6907.346 | 7601.478 | 118034.577 | 475.927 |
| 132 | 22 | 0.331 | 0.022 | 0.050 | 4.986 | 0.010 | 0.010 | 0.358 | 4.633 | 0.010 | 0.103 | 0.322 | 107.811 | 0.010 | 0.010 | 101.706 | 0.001 | 0.263 | 2.028 | 0.353 | 272293.101 | 252.560 | 7549.776 | 8068.083 | 123708.470 | 658.885 |
| 133 | 84 | 0.592 | 192.016 | 193.708 | 5.478 | 0.010 | 0.010 | 0.270 | 1.640 | 0.010 | 0.043 | 0.144 | 136.806 | 0.009 | 0.070 | 236.029 | 0.001 | 1.698 | 1974.388 | 36.903 | 249745.527 | 361.865 | 8296.475 | 6421.555 | 109599.731 | 589.477 |
| 134 | 84 | 0.415 | 7.440 | 0.425 | 4.709 | 0.010 | 0.010 | 0.445 | 0.651 | 0.010 | 0.046 | 0.088 | 121.659 | 0.002 | 0.010 | 205.256 | 0.001 | 0.473 | 2.909 | 31.422 | 250392.254 | 317.946 | 8149.044 | 6640.788 | 111170.843 | 385.819 |
| 135 | 58 | 0.322 | 2.504 | 0.202 | 4.478 | 0.010 | 0.010 | 0.560 | 3.882 | 0.010 | 0.044 | 0.240 | 135.421 | 0.005 | 0.014 | 201.993 | 0.001 | 0.787 | 3.899 | 15.962 | 245601.750 | 350.407 | 8474.423 | 6984.014 | 110318.573 | 1026.467 |
| 136 | 55 | 0.391 | 3.045 | 1.211 | 5.758 | 0.010 | 0.010 | 0.281 | 0.507 | 0.010 | 0.041 | 0.213 | 117.835 | 0.001 | 0.039 | 75.710 | 0.385 | 0.333 | 1.374 | 4.062 | 263979.776 | 205.752 | 8049.931 | 7861.218 | 118082.599 | 596.225 |
| 137 | 20 | 0.002 | 2.813 | 0.050 | 4.196 | 0.010 | 0.010 | 0.231 | 0.763 | 0.010 | 0.052 | 0.637 | 96.482 | 0.010 | 0.026 | 148.854 | 0.001 | 0.179 | 2.368 | 0.692 | 244991.216 | 211.243 | 8085.017 | 6948.785 | 111048.166 | 568.909 |
| 138 | 41 | 0.184 | 1.659 | 0.399 | 4.580 | 0.010 | 0.010 | 0.504 | 0.512 | 0.010 | 0.058 | 0.569 | 126.068 | 0.010 | 0.010 | 151.737 | 0.001 | 0.747 | 2.028 | 2.209 | 254721.825 | 261.262 | 8400.044 | 7413.287 | 115254.234 | 465.003 |
| 139 | 37 | 0.180 | 1.111 | 0.149 | 4.234 | 0.010 | 0.010 | 0.291 | 0.400 | 0.010 | 0.058 | 0.476 | 98.914 | 0.010 | 0.142 | 193.757 | 0.001 | 0.759 | 2.021 | 2.344 | 252422.164 | 220.874 | 8024.030 | 7559.496 | 113442.669 | 380.902 |
| 140 | 50 | 0.188 | 1.171 | 0.282 | 4.347 | 0.010 | 0.010 | 1.198 | 1.108 | 0.010 | 0.078 | 0.629 | 113.945 | 0.002 | 0.010 | 165.546 | 0.001 | 1.410 | 3.310 | 4.838 | 241436.123 | 198.515 | 8108.427 | 7319.513 | 107486.864 | 838.612 |
| 141 | 26 | 0.202 | 0.493 | 0.189 | 5.517 | 0.010 | 0.010 | 0.505 | 0.958 | 0.010 | 0.051 | 0.274 | 103.028 | 0.010 | 0.026 | 87.060 | 0.001 | 0.518 | 1.240 | 0.561 | 266068.183 | 241.069 | 6033.974 | 7732.126 | 114518.409 | 191.621 |
| 142 | 19 | 0.257 | 0.139 | 0.050 | 5.115 | 0.010 | 0.010 | 0.487 | 0.987 | 0.010 | 1.470 | 0.328 | 92.143 | 0.010 | 0.027 | 108.718 | 0.001 | 0.301 | 1.666 | 0.295 | 271736.410 | 262.614 | 6268.136 | 7262.271 | 117038.126 | 676.620 |
| 143 | 41 | 0.229 | 2.335 | 0.505 | 4.037 | 0.010 | 0.010 | 0.565 | 1.047 | 0.010 | 0.076 | 0.694 | 119.320 | 0.004 | 0.060 | 144.562 | 0.001 | 0.737 | 2.528 | 1.401 | 251974.794 | 242.126 | 5519.020 | 6929.830 | 108334.233 | 490.608 |
| 144 | 29 | 0.496 | 0.030 | 0.050 | 4.730 | 0.010 | 0.010 | 1.310 | 4.220 | 0.010 | 0.061 | 0.322 | 141.444 | 0.007 | 0.059 | 113.907 | 0.001 | 0.969 | 3.331 | 0.833 | 263161.955 | 345.290 | 5139.621 | 6768.301 | 116413.245 | 459.476 |
| 145 | 36 | 0.093 | 0.216 | 0.392 | 5.283 | 0.010 | 0.002 | 0.669 | 2.064 | 0.010 | 0.077 | 0.474 | 132.516 | 0.010 | 0.057 | 156.354 | 0.001 | 0.614 | 6.130 | 1.832 | 254523.302 | 248.490 | 7794.976 | 7127.068 | 110367.144 | 741.464 |
| 146 | 65 | 17.731 | 1.996 | 0.050 | 5.049 | 0.010 | 0.011 | 0.427 | 1.525 | 0.010 | 0.050 | 0.327 | 115.509 | 0.010 | 0.061 | 315.935 | 0.001 | 0.481 | 1.872 | 16.072 | 250214.910 | 211.291 | 7846.665 | 7591.420 | 110815.351 | 352.044 |
| 147 | 21 | 0.102 | 0.030 | 0.050 | 5.182 | 0.010 | 0.007 | 0.324 | 2.978 | 0.010 | 0.072 | 0.055 | 103.330 | 0.010 | 0.010 | 124.782 | 0.001 | 0.051 | 2.333 | 0.416 | 260563.851 | 210.102 | 5977.318 | 7521.955 | 115590.228 | 257.261 |
| 148 | 30 | 0.002 | 0.253 | 0.227 | 3.857 | 0.010 | 0.010 | 0.311 | 0.298 | 0.010 | 0.057 | 0.205 | 123.955 | 0.004 | 0.010 | 81.147 | 0.001 | 0.158 | 1.876 | 0.789 | 230255.391 | 174.182 | 7725.215 | 7005.039 | 99777.464 | 660.782 |
| 149 | 30 | 0.227 | 2.447 | 2.197 | 4.597 | 0.010 | 0.038 | 0.243 | 1.295 | 0.010 | 0.101 | 0.640 | 114.359 | 0.007 | 0.010 | 106.476 | 0.001 | 0.301 | 3.065 | 1.426 | 239373.743 | 192.698 | 8979.409 | 7310.796 | 107039.763 | 768.882 |
| 150 | 25 | 0.190 | 0.030 | 7.200 | 0.920 | 0.010 | 0.010 | 0.450 | 11.200 | 0.010 | 0.010 | 0.010 | 92.800 | 0.010 | 0.010 | 161.000 | 0.001 | 0.080 | 0.090 | 5.500 | 222058.000 | 270.000 | 5784.000 | 5418.000 | 10950.000 | 450.117 |
|  |  |  |  |  |  |  |  |  |  |  |  |  |  |  |  |  |  |  |  |  |  |  |  |  |  |  |
